# Suppression of local inflammation via galectin-anchored indoleamine 2,3-dioxygenase

**DOI:** 10.1038/s41551-023-01025-1

**Published:** 2023-05-01

**Authors:** Evelyn Bracho-Sanchez, Fernanda G. Rocha, Sean K. Bedingfield, Brittany D. Partain, Sabrina L. Macias, Maigan A. Brusko, Juan M. Colazo, Margaret M. Fettis, Shaheen A. Farhadi, Eric Y. Helm, Kevin Koenders, Alexander J. Kwiatkowski, Antonietta Restuccia, Bethsymarie Soto Morales, Arun Wanchoo, Dorina Avram, Kyle D. Allen, Craig L. Duvall, Shannon M. Wallet, Gregory A. Hudalla, Benjamin G. Keselowsky

**Affiliations:** 1 J. Crayton Pruitt Family Department of Biomedical Engineering, University of Florida, Gainesville, FL, USA.; 2 Department of Oral Biology, College of Dentistry, University of Florida, Gainesville, FL, USA.; 3 Department of Biomedical Engineering, Vanderbilt University, Nashville, TN, USA.; 4 Department of Anatomy and Cell Biology, College of Medicine, University of Florida, Gainesville, FL, USA.; 5 H. Lee Moffitt Cancer Center and Research Institute, Tampa, FL, USA.; 6 Division of Oral and Craniofacial Health Sciences, Adams School of Dentistry, Department of Microbiology and Immunology, School of Medicine, University of North Carolina at Chapel Hill, Chapel Hill, NC, USA.; 7 These authors contributed equally: Evelyn Bracho-Sanchez, Fernanda G. Rocha, Sean K. Bedingfield, Brittany D. Partain, Sabrina L. Macias.

## Abstract

The treatment of chronic inflammation with systemically administered anti-inflammatory treatments is associated with moderate-to-severe side effects, and the efficacy of locally administered drugs is short-lived. Here we show that inflammation can be locally suppressed by a fusion protein of the immunosuppressive enzyme indoleamine 2,3-dioxygenase 1 (IDO) and galectin-3 (Gal3). Gal3 anchors IDO to tissue, limiting the diffusion of IDO-Gal3 away from the injection site. In rodent models of endotoxin-induced inflammation, psoriasis, periodontal disease and osteoarthritis, the fusion protein remained in the inflamed tissues and joints for about 1 week after injection, and the amelioration of local inflammation, disease progression and inflammatory pain in the animals were concomitant with homoeostatic preservation of the tissues and with the absence of global immune suppression. IDO-Gal3 may serve as an immunomodulatory enzyme for the control of focal inflammation in other inflammatory conditions.

Chronic inflammation, characterized by professional immune cell and resident tissue cell interactions, irreversibly damages tissues of the body and is an associated risk factor for a host of diseases such as cardiovascular disease, diabetes and cancer^1,2^. A major challenge in the treatment of chronic inflammatory diseases is the development of therapeutics to safely and specifically direct resolution of inflammation in a site-specific manner^1^. Anti-inflammatory drugs such as glucocorticoids are pleiotropic, non-specifically affecting numerous pathways^1^ and are consequently accompanied by issues of toxicity, resistance and a wide array of serious adverse effects such as infection and defective wound healing^3^. Additionally, systemic immune modulation leads to disease states such as hypertension, osteoporosis, obesity, cataracts and diabetes^3^. Biologic immunosuppressive drugs functioning through either cytokine blockade, cell depletion or cell surface receptor blockade provide improved specificity and can effectively modulate immune responses to halt disease progression in certain, but not all patients^4^. However, such treatments can also increase susceptibility to infections and disrupt tissue homoeostasis, leading to cancer, exacerbation of congestive heart failure and neurologic events, among other pathologies^4^. Critically, each of these therapeutics require life-long continual use and clinical options to resolve chronic inflammation and restore tissue homoeostasis remain to be developed^5^.

The capability to direct cellular metabolism to programme immune responses has recently emerged as a new avenue for therapeutic immunomodulation^6^. Catabolism of the essential amino acid tryptophan (Trp) by the cytosolic enzyme indoleamine 2,3-dioxygenase 1 (IDO) and the resultant production of kynurenine metabolites is a general regulator of inflammation in response to sterile and pathogenic inflammatory stimuli, acting on both innate and adaptive immune cells^7,8^. IDO catabolism of Trp is also a contributing factor to promoting fetal tolerance in pregnancy, warding off autoimmunity and avoiding immune elimination in some forms of cancer^9^. Trp insufficiency via IDO activates the metabolic stress sensor general control nonderepressible 2 (GCN2) to regulate immune cell cycle^10^, while kynurenine pathway metabolites activate anti-inflammatory programmes, such as kynurenine binding to the aryl hydrocarbon receptor (AHR)^11^. IDO expression in immune cells such as macrophages and dendritic cells has been demonstrated to suppress T cell activation and proliferation while activating and promoting phenotypic maintenance of regulatory T cells^12–14^. Additionally, the terminal product of the kynurenine pathway, nicotinamide adenine dinucleotide (NAD+), regulates innate immunity function in macrophages during aging and inflammation^15^. In total, actions of IDO serve as a key mechanism maintaining homoeostasis, suppressing autoimmunity and shutting down excess inflammation^9,10^. Informed by these data, we envisioned therapeutic delivery of exogenous IDO as a regulator of chronic inflammatory diseases.

A major obstacle to treating localized tissue inflammation without systemic immunosuppression is the need to accumulate therapeutics at the intended site of action. Conceptually aligned with growth factors and antibodies engineered to bind extracellular matrix proteins for localized tissue retention^16–19^, we recently demonstrated the utility of model enzymes fused to galectin-3 (Gal3), a carbohydrate-binding protein, as a generalizable means to restrict enzyme diffusion via binding to tissue glycans^20^. Gal3 binds *N*-acetyllactosamine and other *β*-galactoside glycans, as well as glycosaminoglycans, which are highly abundant in mammalian tissues^21,22^, collectively representing a more universal target than a specific extracellular matrix protein. Thus, Gal3 fusion to enzymes represents a promising approach to retain local enzymatic function at an intended site of action. Building upon this success, we engineered the IDO-Gal3 fusion with the expectation of creating a tissue-anchored IDO administered as a localized anti-inflammatory therapeutic (Fig. 1a,b). Efficacy is described in four distinct inflammatory settings: endotoxin-induced inflammation, psoriasis, periodontal disease and osteoarthritis.

## Results and discussion

### IDO-Gal3 ameliorates skin inflammation

**Fig. 1 |:**
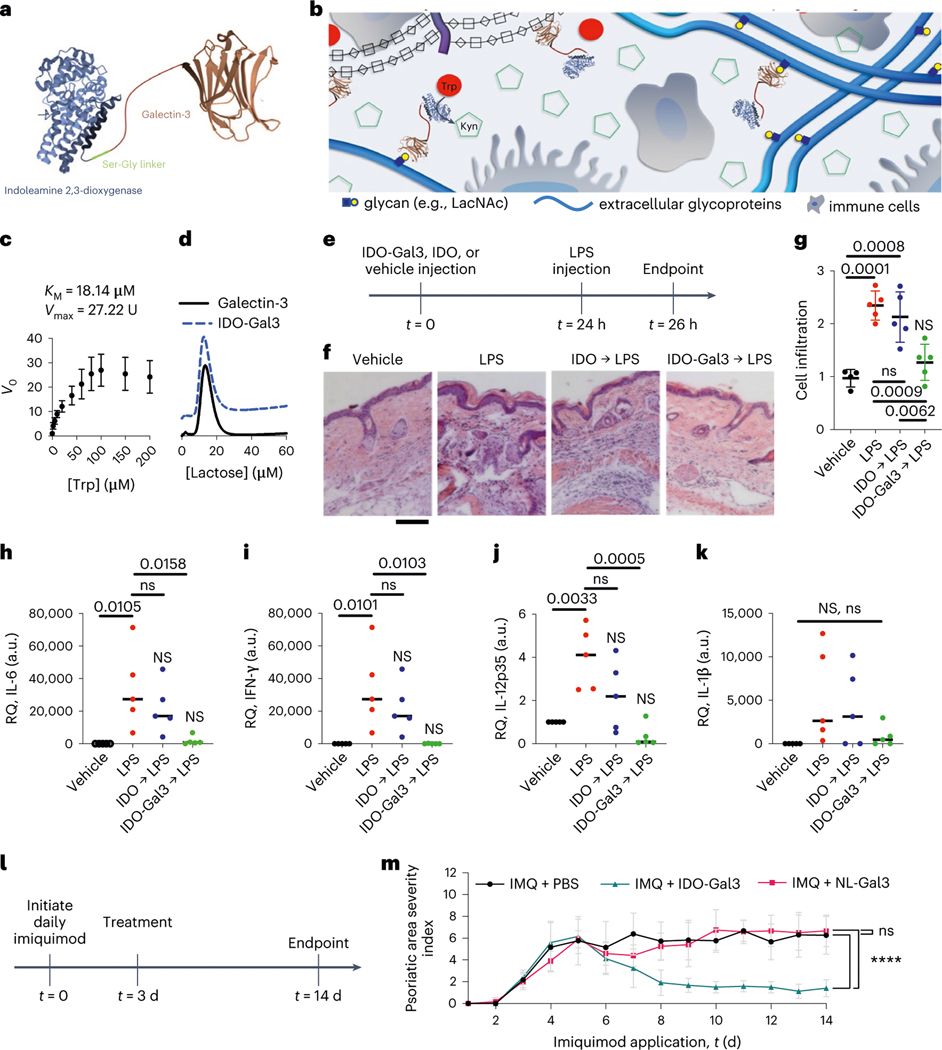
IDO-Gal3 suppresses inflammation. **a**,**b**, Schematic representation of the IDO-Gal3 fusion protein (**a**) and the concept of anchoring IDO at different tissue locations through Gal3-mediated recognition of tissue glycans (**b**). **c**,**d**, Characterization of IDO-Gal3 enzymatic activity (**c**), plotted as initial rate (*V*_0_) vs substrate concentration, where initial and maximum rate (*V*max) are expressed in units, U (pmol(n-formyl kynurenine) min^−1^ pmol^−1^(IDO)), and binding to immobilized lactose (**d**). **e**, Schedule to evaluate anchored IDO suppression of inflammation resulting from local LPS injection. **f**,**g**, Histological evaluation (**f**) of IDO-Gal3 suppression of inflammation induced by injected LPS by enumeration of cell infiltration (**g**). Scale bar, 50 microns. **h**–**k**, Relative quantification (RQ) values of inflammatory gene (IL-6, IFN-γ, IL-12, IL-1β) expression in tissues treated with anchored IDO before challenge with LPS. **l**,**m**, Schedule (**l**) and psoriatic area severity index (**m**) to evaluate anchored IDO suppression of inflammation induced by topical imiquimod. The Gal3-containing protein, NanoLuc^®^ luciferase fusion with Gal3 (NL-Gal3) and lacking the IDO domain, is included as a control. Data shown as mean ± s.e.m. in **c**, and mean ± s.d. in **g** and **m**. Statistical analysis: (**g**–**k**) one-way analysis of variance (ANOVA) with Tukey’s post-hoc. NS indicates no difference compared to vehicle, ns indicates no difference compared to LPS positive control, bar denotes *P* value relative to LPS positive control; *n* = 6. For **g**, *P* = 0.0054, *F* = 6.200. For **h**, *P* = 0.0040, *F* = 6.630. For **i**, *P* = 0.0006, *F* = 10.15. For **j**, *P* = 0.0898, *F* = 2.579. For **m**, Mann-Whitney *U*-test with alpha = 0.05, ****P < 0.0001, *n* = 12.

IDO-Gal3 catalysed the conversion of Trp to kynurenine (Fig. 1c) and bound immobilized lactose with comparable affinity as wild-type Gal3 (Fig. 1d). Subcutaneous (s.c.) injection of IDO-Gal3 prevented inflammation induced by a local lipopolysaccharide (LPS) challenge better than IDO alone (Fig. 1e–k). Histological analysis demonstrated that 24 h pre-treatment with IDO-Gal3 mitigated the cellular infiltration that follows LPS challenge, whereas pre-treatment with unanchored IDO did not (Fig. 1e–g). Naïve tissue cellular infiltration was unaffected by either IDO or IDO-Gal3 s.c. injection, compared to saline vehicle (PBS; Supplementary Fig. 1). Pre-treatment with IDO-Gal3 blocked transcription of the pro-inflammatory cytokines IL-6, IFN-γ and IL-12p35, whereas expression was higher than in vehicle control following pre-treatment with IDO (Fig. 1h–j). IL-1β transcript levels varied considerably following LPS challenge, but the trend suggested that pre-treatment with IDO-Gal3 also suppressed expression of this pro-inflammatory cytokine, whereas pre-treatment with IDO did not (Fig. 1k). Unchallenged naïve tissue gene expression of inflammatory cytokines was unaffected by either IDO or IDO-Gal3 s.c. injection, compared to saline vehicle (PBS; Supplementary Fig. 2). Additionally, s.c. administration of the control protein, NanoLuc fusion with Gal3 (NL-Gal3), lacking the IDO domain had little to no effect on either naïve or LPS-challenged tissue gene expression (Supplementary Fig. 3). This suggested that the Gal3 domain did not play an in vivo immunomodulatory role in the IDO-Gal3 fusion protein. Further in vitro characterization was consistent with no immunomodulation by the Gal3 fusion domain, as the capability of IDO-Gal3 to suppress LPS activation of cultured bone marrow-derived dendritic cells mirrored previous results with wild-type IDO alone, and NL-Gal3 treatment did not alter dendritic cell phenotype (Supplementary Fig. 4).

Local injection of IDO-Gal3 also resolved inflammation in a murine psoriasis model (Fig. 1l,m). Topical daily application of imiquimod over 14 d induced inflammation, which peaked at day 5, as reflected by psoriasis severity clinical score. Vehicle injection did not affect the severity score, which remained elevated for the remaining 14 d period. In contrast, subcutaneous injection of IDO-Gal3 on day 3 led to a dramatic decrease in clinical score by day 8, which remained low for the duration despite continued daily imiquimod challenge. Additionally, administration of the control fusion protein NL-Gal3 demonstrated no effect on psoriasis severity (Fig. 1m), corroborating the indication that the Gal3 domain does not play an immunomodulatory role in the therapeutic response of IDO-Gal3. Consistent with this, our previous work^20,23–25^ shows that fusion of proteins onto the N terminus of Gal3, such as NanoLuc, GFP or galectin-1, knocks out Gal3 signalling activity, probably because its ability to form higher-ordered oligomers is inhibited while glycan-binding affinity is maintained.

Given the capacity for endogenous cellular IDO gene expression (*Ido1*) to serve as positive feedback to further elevate expression^9^, endogenous *Ido1* transcripts were quantified after treatment with IDO-Gal3 in both the LPS and psoriasis models (Supplementary Fig. 5). While both LPS and imiquimod challenge induced endogenous *Ido1* expression as an expected response to inflammation, interestingly, the challenged IDO-Gal3-treated tissues did not, with levels comparable to baseline. These data suggest that exogenous supply of IDO-Gal3 blocked inflammatory signalling and the associated induction of endogenous *Ido1* expression in local tissues; however, low-level endogenous IDO expression induced by IDO-Gal3 in specific cell subsets (for example, dendritic cells either local or in lymphoid tissue) could also potentially contribute to the resolution of inflammation. In sum, s.c. administration of IDO-Gal3 ameliorated skin inflammation both prophylactically and therapeutically, using two different inflammatory insults acting through different specific receptors (CD14/TLR4 for LPS and TLR7 for imiquimod)^26^.

### IDO-Gal3 acts locally and is not systemically suppressive

We recently demonstrated that Gal3 fusion to various enzymes endows glycan-binding affinity that restricts enzyme diffusion in tissues, prolonging retention and localizing enzymatic catalysis^20^. In vivo imaging detected fluorophore-labelled IDO-Gal3 out to 14 d in s.c. hock injection site (Supplementary Fig. 6a). Increased kynurenine levels in surrounding tissue detected via mass spectrometry analysis were consistent with in vivo IDO enzymatic function (Supplementary Fig. 6b). Further, bioluminescence imaging of anchored reporter enzyme, the fusion of NanoLuc luciferase and Gal3 (NL-Gal3), revealed it was retained at high levels compared with unanchored NanoLuc, and suggested that NL-Gal3 s.c. injected into hock was largely restricted to the injection site, although some lower enzyme activity was also detected in the adjacent tibial region, in plasma and the excretory pathways of the urine and faeces (Supplementary Fig. 7). To determine whether localized IDO-Gal3 induced systemic immunosuppression (Supplementary Fig. 6c–k), 120 h after injection, mice were subjected to LPS challenge at ipsilateral and contralateral sites, and separately, an oral *Listeria monocytogenes* infection challenge. Prophylactic administration of IDO-Gal3 in the ipsilateral hock blocked LPS-induced inflammatory cell infiltration even 120 h after pre-treatment, whereas infiltrate was elevated at the contralateral LPS challenge site, suggesting the inflammatory blockade was localized to the ipsilateral site (Supplementary Fig. 6c,d). Administering IDO-Gal3 into the s.c. hock did not alter *L. monocytogenes* clearance in the liver or spleen when compared to vehicle control (Supplementary Fig. 6e–g), which indicated maintenance of a globally functioning immune system concurrent with the tissue-localized suppression. In corroboration with prolonged reduction in cellular infiltration data at 120 h after pre-treatment with IDO-Gal3 (Supplementary Fig. 6c,d), expression of IL-6 and IL-1β at the ipsilateral LPS challenge site (Supplementary Fig. 6h,i) was suppressed; expression of IL-12p35, IL-12p40 and IFN-γ (Supplementary Fig. 8) was also suppressed, preventing increased plasma IL-6 protein (Supplementary Fig. 6j) and maintaining baseline plasma IL-1β levels (Supplementary Fig. 6k) detected in serum by mass spectrometry. Collectively, these observations suggest that tissue-anchored IDO-Gal3 induced potent local suppression of inflammation without globally suppressing immune function.

### IDO-Gal3 averts and inhibits periodontal disease progression

**Fig. 2 |:**
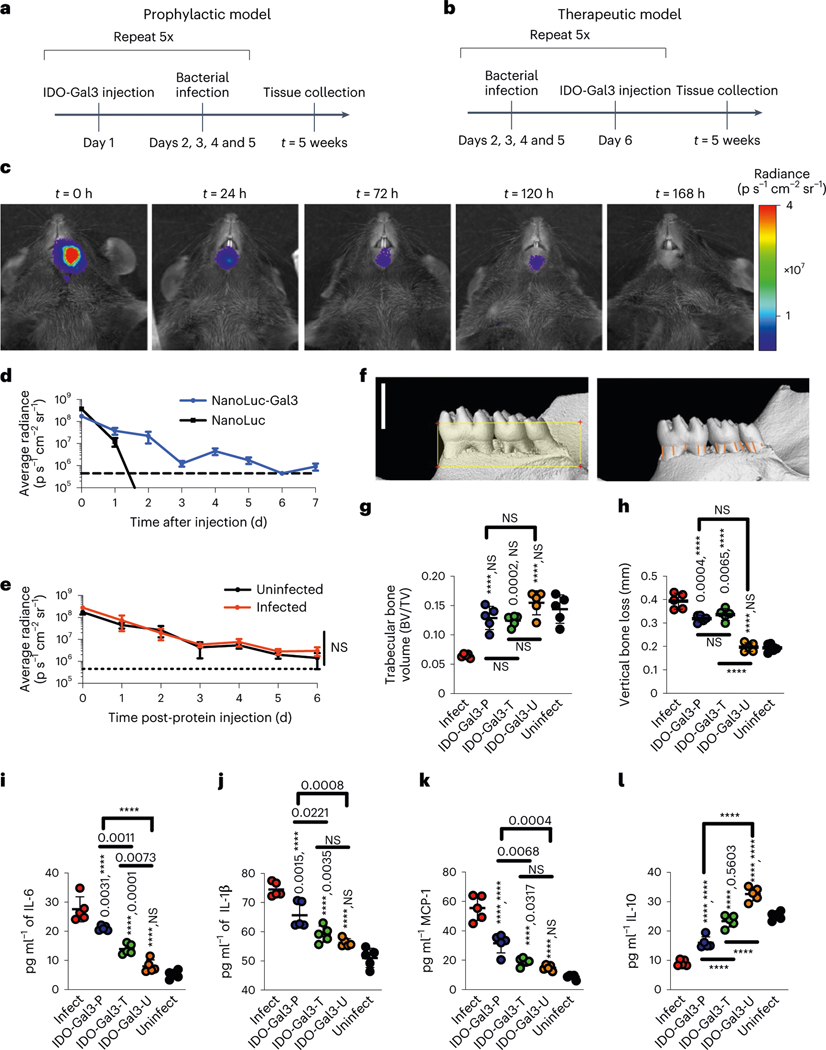
IDO-Gal3 prevents and inhibits periodontal disease progression. **a**,**b**, Efficacy was investigated using a polymicrobial mouse model of periodontal disease and either prophylactic (**a**) or therapeutic (**b**) administration. **c**,**d**, Anchored-fusion reporter enzyme NanoLuc-Gal3 (NL-Gal3) injected submandibularly was retained locally for 120 h, by in vivo imaging (**c**), in contrast to unanchored NanoLuc which diffused away from the injection site (**d**, dashed line represents baseline). **e**, Infection state did not alter submandibular retention time of NL-Gal3; dotted line represents baseline. **f**–**h**, Representative images of micro-CT analysis (**f**) quantifying trabecular bone volume (**g**) and vertical bone loss (**h**), by measuring the cementoenamel junction. In **f**: scale bar, 1.5 mm; the yellow box represents the standardized ROI of 5.5 mm^3^, based on 1.5 × 4.0 × 1.0 mm (vertical or cervico-apical × horizontal or mesio-distal × lateral or buccal-palatal); the orange lines indicate the measured distances between the CEJ and the alveolar bone crest (*n* = 12). Submandibular injection of IDO-Gal3 blocked mandibular bone loss in prophylactic (IDO-Gal3-P) administration and halted mandibular bone loss in therapeutic (IDO-Gal3-T) administration. **i**–**k**, IDO-Gal3 reduced gingival inflammatory protein levels of IL-6 (**i**), IL-1β (**j**) and MCP1 (**k**), with a more pronounced effect from therapeutic administration (IDO-Gal3-T). **l**, IDO-Gal3 induced the expression of IL-10 protein, restoring levels closer to that of the uninfected control (Uninfect). IDO-Gal3 administered to uninfected mice (IDO-Gal3-U) had negligible effect on mandibular bone and production of inflammatory cytokines while increasing levels of IL-10 and to a smaller extent, MCP1. Data shown as mean ± s.e.m. (**d** and **e**) and mean ± s.d. (**g**–**l**). Statistical analysis: (**e**) Student’s *t*-test; *n* = 5; (**g**–**l**) one-way ANOVA with Tukey’s post-hoc test, *n* = 5. For (**g**), *P* < 0.0001, *F* = 20.41. For **h**, *P* < 0.0001, *F* = 75.53. For **i**, *P* < 0.0001, *F* = 73.69. For **j**, *P* < 0.0001, *F* = 44.24. For **k**, *P* < 0.0001, *F* = 66.63. For **l**, *P* < 0.0001, *F* = 122.8. For **g**–**l**, unless otherwise specified, pairwise *P* values from Tukey’s post-hoc analysis are reported as ‘X, Y’, where X represents comparison to ‘Infect’ and Y represents comparison to ‘Uninfect’; ****P < 0.0001.

Periodontal disease is a class of non-resolving chronic inflammatory diseases resulting from localized mucosal inflammation and osteoclast activation, and leading to soft (mucosa) and hard (bone) tissue destruction^27^. Evaluated with both a prophylactic and therapeutic administration scheme in a polymicrobial periodontal disease mouse model^28,29^ (Fig. 2a,b), submandibular injection of IDO-Gal3 was locally retained, suppressed mucosal inflammation and spared alveolar bone loss (Fig. 2c–l). In vivo imaging of anchored reporter NL-Gal3 demonstrated gingival retention for 120 h (Fig. 2c,d), whereas tissue localization for the unanchored NL could not be measured after 36 h (Fig. 2d). Retention time was not altered in the presence or absence of polymicrobial infection (Fig. 2e). The primary outcome of periodontal disease, mandibular bone loss, was quantified by micro-computed tomography (micro-CT) analysis. When administered prophylactically (IDO-Gal3-P), submandibular injection prevented bone loss, and IDO-Gal3 inhibited further bone loss when administered therapeutically (IDO-Gal3-T; Fig. 2f). Specifically, loss of trabecular bone volume (Fig. 2g) and thickness (Supplementary Fig. 9), along with vertical bone loss (Fig. 2h), were prevented by either prophylactic or therapeutic administration of IDO-Gal3. IDO-Gal3 also inhibited gingival inflammation as measured by protein levels of cytokines IL-6 (Fig. 2i) and IL-1β (Fig. 2j), and chemokine MCP1 (Fig. 2k). In contrast, IL-10 production was largely maintained through IDO-Gal3 treatment (Fig. 2l), whereby levels were closer to those of uninfected mice, suggesting a return toward homoeostatic control. A more pronounced diminution of bone loss and suppression of inflammation was observed upon therapeutic administration of IDO-Gal3 when compared to prophylactic administration (Fig. 2h–k), where the inflammatory cytokines IL-12p70, IP-10, KC-17, MIP-2 and IL-33 were most affected (Supplementary Fig. 10). IDO-Gal3 administered to uninfected mice (IDO-Gal3-U) had negligible effect on mandibular bone and production of inflammatory cytokines; however, increased levels of IL-10 and to a smaller extent, MCP1, were observed (Fig. 2j–l). Furthermore, IDO-Gal3 administration did not affect the amount of either *Porphoromonas gingivalis* or *Aggregatibacter actinomycetemcomitans* recovered from the gingiva, regardless of prophylactic or therapeutic administration scheme (Supplementary Fig. 11). Together these data indicate that IDO-Gal3 addresses the clinical therapeutic requirements for treatment of periodontal disease: localized regulation of inflammation without enhancing bacterial accumulation, and prevention of continued mandibular bone loss even after disease progression has begun.

### IDO-Gal3 prevents post-traumatic osteoarthritis

**Fig. 3 |:**
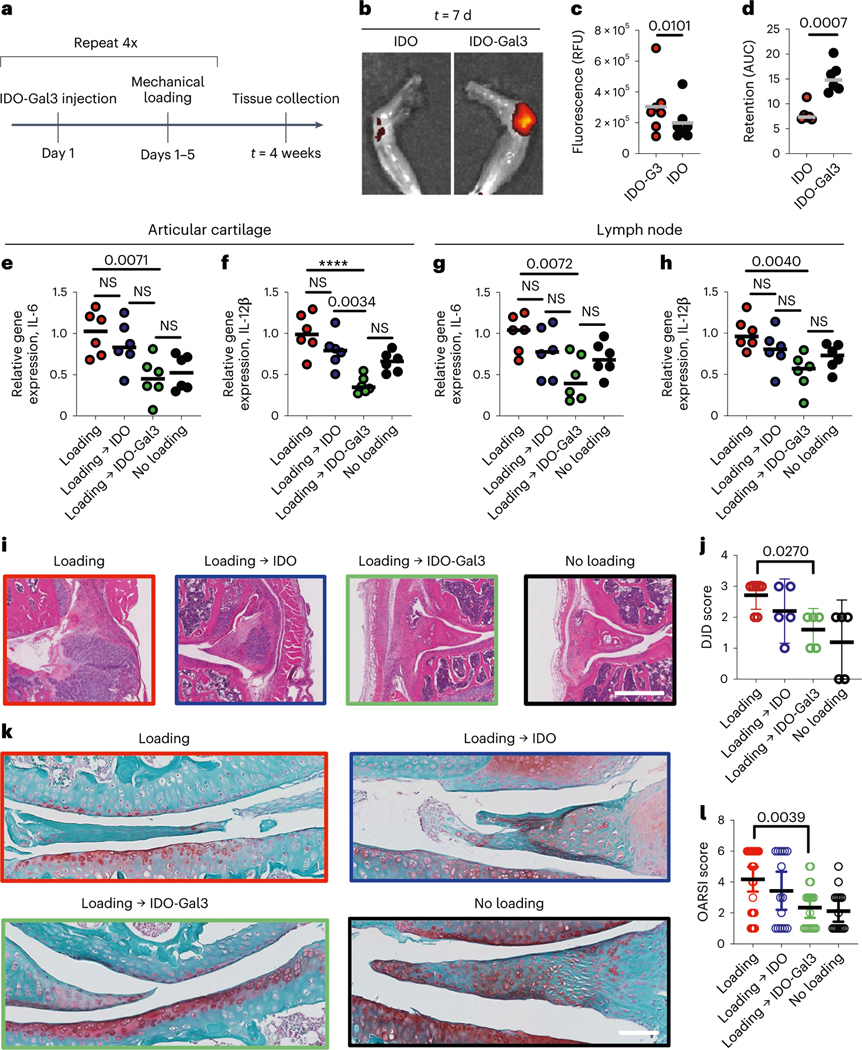
IDO-Gal3 protects against upregulation of inflammatory gene expression and joint structural changes from load-induced osteoarthritis. **a**, A 4-week cyclic mechanical overloading PTOA mouse model was utilized in mice treated weekly with intra-articular IDO-Gal3 injections. **b**,**c**, Knee explant fluorescence imaging illustrating (**b**) and quantifying (**c**) day 7 retention of fluorescently labelled IDO and IDO-Gal3 administered after 2 weeks of mechanical loading. **d**, AUC pharmacokinetic analysis from daily fluorescent imaging over 7 d. **e**–**h**, Gene expression of cytokines IL-6 and IL-12p40 was measured by quantitative PCR in combined joint tissue of synovial wall and articular surface (**e**,**f**) and draining lymph nodes (**g**,**h**). **i**,**j**, H&E-stained joint histology on knees highlighting synovial inflammation and thickening (**i**) and DJD scoring of joint disease progression (**j**). **k**,**l**, Safranin O histological stain highlighting structure changes and proteoglycan content of the articular cartilage (**k**) and OARSI scoring of cartilage structure (**l**). Data in **j** and **l** shown as mean ± 95% C.I. Statistical analysis: (**c**,**d**) Student’s *t*-test, *n* = 6. For **e**–**h**: one-way ANOVA with Tukey’s post-hoc test; *n* = 6. For **e**, *P* = 0.0035, *F* = 6.303. For **f**, *P* < 0.0001, *F* = 12.92, ****P < 0.0001. For **g**, *P* = 0.0128, *F* = 4.639. For **h**, *P* = 0.0069, *F* = 5.403. For **j** and **l**, Brown-Forsythe and Welch ANOVA; *n* = 6. For **j**, *P* = 0.0325, F* = 4.173. For **l**, *P* = 0.0012, F* = 6.083. *P* values reported for statistically different groups; all other groups, NS.

Post-traumatic inflammatory osteoarthritis (PTOA), a form of osteoarthritis (OA), commonly arises after a ligament or meniscus tear, or following repeated overloading of a joint. A cyclic mechanical overloading PTOA mouse model^30,31^ was utilized to test for the ability of IDO-Gal3 to reduce load-induced inflammatory joint damage. Intra-articular injection of IDO-Gal3 was locally retained, suppressed inflammation and spared joint tissue destruction (Fig. 3). Cyclic mechanical loading was applied to knees of aged mice (Fig. 3a and Supplementary Fig. 12), with mechanical damage and consequent inflammation resulting in synovial inflammation and cartilage degradation. In a retention study, fluorescently labelled IDO or labelled IDO-Gal3 was administered after 2 weeks of initial joint mechanical loading. Imaging at 24 h (Supplementary Fig. 13) and day 7 post-injection revealed that retention of labelled IDO-Gal3 in the joint was significantly higher than unanchored IDO, visualized in explanted knees (Fig. 3b) and quantified (Fig. 3c) by ex vivo image analysis. The pharmacokinetic area-under-curve (AUC), calculated from daily intravital imaging over 7 d, was approximately 2-fold higher for IDO-Gal3 than IDO alone (Fig. 3d). Weekly intra-articular administration of IDO-Gal3 blocked gene expression of inflammatory cytokines IL-6 and IL-12p40 in combined joint tissue (synovial wall and articular surface; Fig. 3e,f) and draining lymph nodes (Fig. 3g,h), whereas IDO alone did not. In contrast, expression of neither MMP13 nor TNF-α was modulated at either location (Supplementary Fig. 14). Critically, IDO-Gal3 reduced load-induced histological changes, as scored by a treatment-blinded histopathologist using both total joint health score (degenerative joint disease (DJD) score) (Fig. 3i,j) and articular cartilage score (OARSI) (Fig. 3k,l). Additional histological specimens support these trends (Supplementary Figs. 15–18; scoring criteria description provided in Supplementary Figs. 19 and 20). In sum, Gal3 fusion provided local joint retention, and IDO-Gal3 potently reduced inflammatory gene expression and mechanical overloading-induced structural changes in the joint.

We note that multiple injections of human IDO-Gal3 in mice led to the emergence of some anti-IDO-Gal3 antibodies (Supplementary Fig. 21), which is not unreasonable given the host mismatch; however, these antibodies did not emerge until after ~3 injections and did not diminish efficacy in multiple injection models (osteoarthritis and periodontal disease) when compared to single injection models (LPS inflammation and psoriasis).

### IDO-Gal3 reduces inflammation in a model of established PTOA

**Fig. 4 |:**
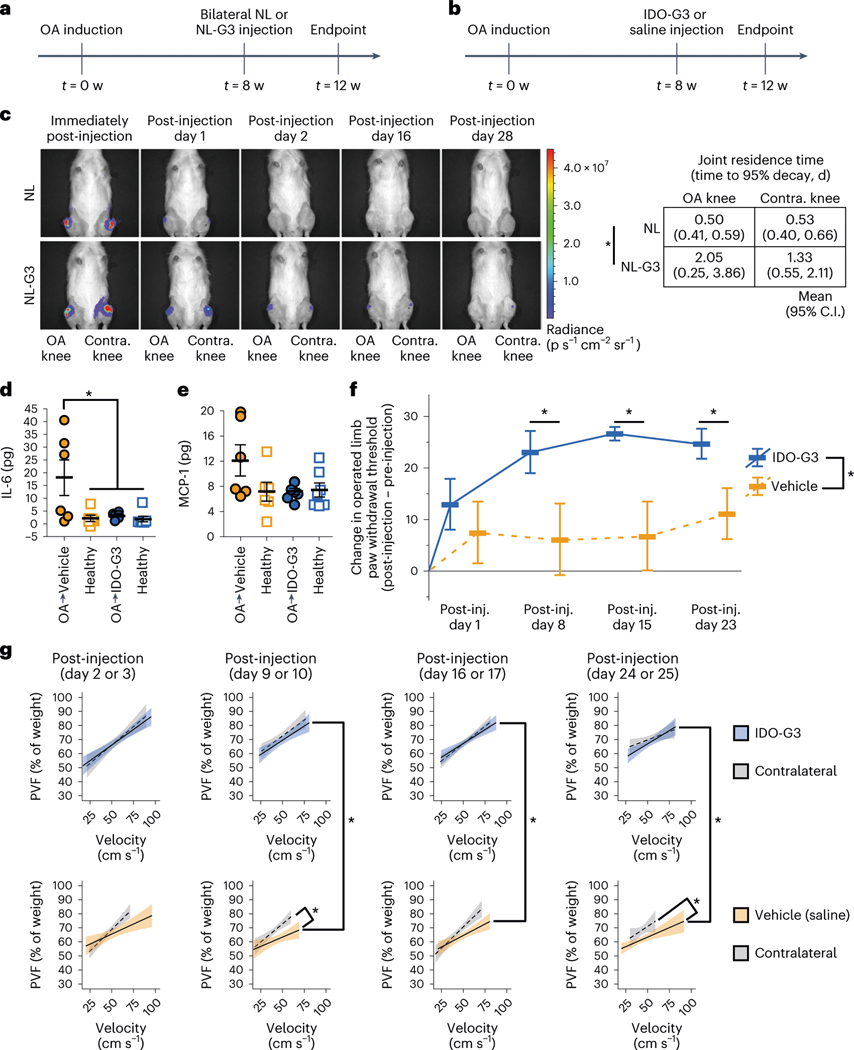
IDO-Gal3 reduces inflammation, decreases tactile hypersensitivity and improves gait after established osteoarthritis. **a**, Following a surgically simulated meniscal tear in the rat, clearance of NL-Gal3 was assessed 8 weeks after accrued joint damage. **b**, Using the same model and experimental design, the effects of IDO-Gal3 on OA-associated inflammation and symptoms were evaluated. **c**, Luminescence imaging (left) demonstrated that NL-Gal3 is retained in both OA-affected and healthy joints longer than unconjugated NL, with significantly longer times to 95% decay (right) in NL-Gal3 injected knees. **d**, At 28 d after injections in OA-affected joints, saline-treated knees had elevated levels of IL-6 (*P* = 0.0021, *t* = 3.4689, d.f. = 23.3), while IDO-Gal3 levels were comparable to healthy contralateral knees. **e**, Intra-articular levels of MCP1 (that is, CCL2) followed a similar profile, but CCL2 was not significantly elevated in saline-treated knees for this study (*P* = 0.09, *F* = 3.41, (d.f., d.f.) = (1, 24)). **f**, Relative to saline-treated animals, IDO-Gal3-treated animals had improvements in tactile sensitivity levels across time. Raw data are shown in Supplementary Fig. 22. **g**, IDO-Gal3-treated animals had similar weight distribution between their OA-affected and contralateral limb, while saline-treated animals had significant off-loading of the OA-affected limb. Raw data are shown in Supplementary Fig. 23. *n* = 6 for OA-vehicle and healthy control in **d** and **e**; *n* = 7 for OA-IDO-G3 and its healthy control in **d** and **e**; *n* = 7 for **f**. In **d**–**f**, data are presented as mean ± 95% C.I. Data in **d** and **e** were analysed with a linear mixed effects model, treating limb and treatment group as fixed factors and animal as a random effect. If indicated by an ANOVA on the fixed effects, multiple comparisons of least squared means estimates were corrected for compounding type 1 errors via a Tukey’s HSD correction. Data in **f** were analysed with a repeated measures ANOVA with post-hoc Tukey’s HSD adjustment for compounding type 1 errors. In **f**: *P = 0.0244, *F* = 6.8883, (d.f., d.f.) = (1, 11). Specific differences between IDO-G3-treated and vehicle-treated knees at each week are as follows: Week 9: *P* = 0.013, *t* = 2.61, d.f. = 41; Week 10: *P* = 0.004, *t* = 3.07, d.f. = 41; and Week 11: *P* = 0.041, *t* = 2.11, d.f. = 41. In **g**: error bands represent 95% C.I. of the population mean; behavioural data were evaluated using a linear mixed effects model treating animal identifiers as a random effect across time. In **g** column 2, vehicle-treated differed from its contralateral control (*P* = 0.002, *t* = 3.099, d.f. = 774) and IDO-G3-treated differed from vehicle-treated (*P* = 0.007, *t* = 2.73, d.f. = 260); in column 3, IDO-G3-treated differed from vehicle-treated (*P* = 0.008, *t* = 2.72, d.f. = 131); and in column 4, vehicle-treated differed from its contralateral control (*P* = 0.004, *t* = 2.88, d.f. = 773) and IDO-G3-treated differed from vehicle-treated (*P* = 0.016, *t* = 2.42, d.f. = 225).

Osteoarthritis pain and disability are associated with chronic inflammation in an irreparably damaged joint^32^. To investigate a single-dose IDO-Gal3 treatment in established OA, a surgically induced PTOA rat model was utilized, where OA developed over 8 weeks of accrued joint damage following transection of the medial collateral ligament and medial meniscus (MCLT + MMT)^33,34^. Intra-articular injection of IDO-Gal3 was locally retained, suppressed joint inflammation, reduced OA-associated pain and improved rat gait (Fig. 4). Administered into both OA and contralateral healthy joints (Fig. 4a), luminescence of the NL-Gal3 anchored reporter was measurable over multiple days post-injection, while unanchored NL was not found in the joint after 1 d (Fig. 4c). Joint residence times (95% decay) for NL-Gal3 were 2 d in OA joints and 1.3 d in healthy joints, which is up to 4-fold longer compared with 0.5 d for NL in both OA and healthy joints (Fig. 4c right). Injection of IDO-Gal3 at 8 weeks post-surgery (Fig. 4b) blocked local inflammatory cytokine production, and furthermore reversed OA-associated tactile sensitivity and compensatory gait (Fig. 4d–g). Treatment with IDO-Gal3 reduced protein production of inflammatory cytokine IL-6 in OA joints to levels found in healthy joints (Fig. 4d). Additionally, local levels of inflammatory chemokine MCP1 followed this same trend (*P* = 0.09; Fig. 4e). Tactile sensitivity as a metric of OA-associated pain was quantified over 3 weeks using von Frey monofilaments (Supplementary Fig. 22, absolute values). Plotted as the change in paw withdrawal threshold (post-injection − pre-injection), treatment with IDO-Gal3 reversed limb hypersensitivity by post-injection day 8, persisting through day 23, representing a 2-fold improvement, while saline vehicle injection provided only marginal improvement (Fig. 4f). Rodent gait was evaluated by simultaneous high-speed videography and force plate recordings^35^. IDO-Gal3-treated rats utilized faster walking speeds on post-injection day 16 and tended to walk faster on post-injection day 23 (Supplementary Fig. 23a,b). Notably, peak vertical force plotted as a function of velocity demonstrated that a single IDO-Gal3 injection corrected imbalanced weight distribution in OA-affected animals, where dynamic loading on IDO-Gal3-treated limbs was indistinguishable from that of healthy contralateral controls throughout the study duration (Fig. 4g top row and Supplementary Fig. 23c). This contrasted with the distinct loading imbalance between saline-treated and contralateral limbs (Fig. 4g bottom row and Supplementary Fig. 23c). Notably, IDO-Gal3 and saline treatments were substantially different at post-injection days 9, 16 and 24. Altogether, these data indicate potent amelioration of established OA inflammation, along with concomitant improvement in OA symptoms.

In sum, the IDO-Gal3 tissue-anchored enzyme fusion represents a new class of anti-inflammatory protein therapeutic with broad potential implications for better treatment outcomes and reduced systemic side effects for a broad variety of localized inflammatory diseases. The cytosolic immunomodulatory enzyme IDO was conceived as an exogenously functioning and locally deliverable protein therapeutic to directly manipulate metabolism of the essential amino acid tryptophan, acting here as a systemically available prodrug. Fusion with Gal3 prolonged enzyme localization and retention through binding to glycans, which are ubiquitously expressed across mammalian tissues. The conservation of glycans across species enables translation without requiring redesign for human-specific targets. Administered IDO-Gal3 robustly controlled local inflammation across four distinct inflammatory settings in multiple species and in multiple tissue environments. Treatment restored homoeostasis, preserved tissue integrity and function, and relieved inflammation-associated pain. Administration of IDO-Gal3 uniformly blocked IL-6 in each inflammatory model investigated, among numerous other cytokines and chemokines, and did not induce systemic immunosuppression. Altogether, this work highlights how the galectin-anchored enzyme overcomes the drug delivery obstacle of diffusion of a therapeutic away from the intended site of action, and points to IDO-Gal3 as an anti-inflammatory drug.

## Methods

### Recombinant protein expression and purification

NanoLuc is the tradename of an engineered deep-sea shrimp luciferase variant developed by Promega Corporation^36^. Genes encoding fusion proteins were inserted into pET-21d(+) vectors between NcoI and XhoI sites. IDO-Gal3 genetic and protein sequences are provided in Supplementary Information. Plasmids confer ampicillin resistance and were first transformed into One Shot TOP10 chemically competent *E. coli* (ThermoFisher), selected on Luria-Bertani (LB) ampicillin (50 μg ml^−1^) agar and incubated overnight at 37 °C. Isolated colonies from the plates were subcultured in 5 ml LB ampicillin broth overnight at 37 °C with orbital shaking. Successful transformants were confirmed by Sanger sequencing (Genewiz). Positive DNA sequences were then transformed into the kanamycin B resistant expression strain, Origami B (DE3) *E. coli* (Novagen) and selected on LB ampicillin with kanamycin B (15 μg ml^−1^) agar. Positive clones were picked, banked in LB with 10% w/v glycerol and used to preculture 5 ml of LB ampicillin and kanamycin. Overnight precultures were used to inoculate 1l 2× TY media (16 g tryptone, 10 g yeast extract, 5 g NaCl) with ampicillin and kanamycin B at 37 °C and 225 r.p.m. in an orbital shaker until approximate exponential growth phase (optical density (OD)_600 nm_ = 0.6–0.8). IDO-Gal3 expression cultures were supplemented with 500 μM *δ*-Aminolevulinic acid (Sigma) at the time of inoculation. Recombinant protein expression was triggered using 0.5 mM isopropyl *β*-d-1-thiogalactopyranoside (ThermoFisher) and incubated for 18 h in an orbital shaker at 18 °C. Bacteria were washed and pelleted with PBS via centrifugation (13,180 × *g* at 4 °C for 10 min) with a superspeed centrifuge (ThermoFisher). Afterwards, the pellet was weighed, resuspended in 4 ml PBS per gram pellet with protease inhibitor (ThermoFisher) and disrupted by sonic dismembration (15 s on, 45 s off, 10 min cycling time; ThermoFisher). Following dismembration, lysates were treated with 20 units per gram pellet DNAse I (ThermoFisher) and 800 μg g^−1^ pellet lysozyme (ThermoFisher). The lysate was centrifuged (38,360 × *g* at 4 °C for 15 min) to remove the insoluble fraction and the supernatant was collected by decanting. Supernatant containing soluble recombinant protein was loaded into an *α*-lactose agarose (Sigma) affinity column pre-equilibrated with PBS. Columns were washed with 20 column volumes PBS and recombinant proteins were eluted with 100 mM β-d-lactose (Sigma) prepared in PBS. A final polishing step was performed by 200 kDa size exclusion chromatography on AKTA pure chromatography system (GE Life Sciences) to remove *α*-lactose and further isolate IDO-Gal3. Protein purity was determined by sodium dodecyl sulfate polyacrylamide gel electrophoresis (SDS–PAGE) and Coomassie staining. Endotoxin contaminants were removed by endotoxin removal solution (Sigma) following manufacturer instructions. Endotoxin content was analysed using Chromo-LAL kinetic chromogenic endotoxin quantification assay (Associates of Cape Cod) and determined to be below 0.1 EU ml^−1^ in all stocks.

### IDO enzymatic activity assay

Recombinant human IDO expressed in *E. coli* was purchased from R&D Systems (predicted molecular mass of 46 kDa, a specific activity of >500 pmol min^−1^ μg^−1^ (or for equimolar calculations >29 pmol NFK min^−1^ pmol^−1^ IDO) as measured by its ability to oxidize l-tryptophan to *N*-formyl-kynurenine (NFK)). The specific activity of both proteins, IDO and IDO-Gal3, was measured before experiments to ensure maximal effect at the beginning of the assay, following the IDO manufacturer’s protocol. IDO-Gal3 was reacted in equimolar amounts to IDO in the standard protocol, and activities were compared using the unit pmol NFK min^−1^ pmol^−1^ IDO to more accurately compare activity.

### IDO-Gal3 binding affinity

Affinity of IDO-Gal3 for lactose was determined using affinity chromatography in an AKTA Pure chromatography system (GE Life Sciences) equipped with consumer-packable glass column (GE Life Sciences) packed with *α*-lactose agarose affinity resin (Sigma-Aldrich). Proteins were eluted with a linear gradient of *β*-lactose (Sigma-Aldrich) in phosphate buffer.

### IDO-Gal3 DNA sequence

The sequence used was CCATGGCGCACGCGATGGAAAACAGCTGGACCATCAGCAAAGAGTACCACATTGACGAGGAAGTTGGTTTCGCGCTGCCGAACCCGCAGGAAAACCTGCCGGACTTCTATAACGATTGGATGTTTATCGCGAAGCACCTGCCGGATCTGATTGAGAGCGGCCAGCTGCGTGAGCGTGTGGAAAAACTGAACATGCTGAGCATCGACCACCTGACCGATCACAAGAGCCAACGTCTGGCGCGTCTGGTTCTGGGTTGCATTACGATGGCGTACGTGTGGGGCAAAGGTCACGGCGACGTGCGTAAGGTTCTGCCGCGTAACATCGCGGTTCCGTACTGCCAACTGAGCAAGAAACTGGAACTGCCGCCGATTCTGGTGTATGCGGACTGCGTTCTGGCGAACTGGAAGAAGAAGGACCCGAACAAACCGCTGACCTATGAGAACATGGATGTGCTGTTCAGCTTTCGTGACGGTGATTGCAGCAAGGGCTTCTTTCTGGTGAGCCTGCTGGTTGAAATCGCGGCGGCGAGCGCGATCAAAGTGATTCCGACCGTTTTCAAGGCGATGCAGATGCAAGAGCGTGACACCCTGCTGAAAGCGCTGCTGGAAATCGCGAGCTGCCTGGAGAAGGCGCTGCAGGTGTTTCACCAAATTCACGATCACGTTAACCCGAAAGCGTTCTTTAGCGTGCTGCGTATCTACCTGAGCGGTTGGAAGGGCAACCCGCAGCTGAGCGACGGTCTGGTTTATGAGGGCTTCTGGGAAGATCCGAAAGAGTTTGCGGGTGGCAGCGCGGGTCAGAGCAGCGTGTTCCAATGCTTTGACGTTCTGCTGGGCATTCAGCAAACCGCGGGTGGCGGTCATGCGGCGCAGTTCCTGCAAGATATGCGTCGTTACATGCCGCCAGCGCACCGTAACTTCCTGTGCAGCCTGGAAAGCAACCCGAGCGTGCGTGAGTTTGTTCTGAGCAAAGGTGACGCGGGCCTGCGTGAAGCGTATGATGCGTGCGTGAAGGCGCTGGTTAGCCTGCGTAGCTACCACCTGCAGATCGTTACCAAATATATCCTGATTCCGGCGAGCCAGCAACCGAAAGAAAACAAGACCAGCGAGGACCCGAGCAAACTGGAGGCGAAGGGTACCGGCGGTACCGATCTGATGAACTTTCTGAAGACCGTGCGTAGCACCACCGAGAAGAGCCTGCTGAAAGAGGGTGGATCCGGCGGCGGCAGCGGCGGCAGCGGCGGCAGCGGCGGCGAATTCGCGGACAACTTCAGCCTGCACGATGCGCTGAGCGGTAGCGGTAACCCGAACCCGCAGGGTTGGCCGGGTGCGTGGGGTAACCAACCGGCGGGTGCGGGTGGCTACCCGGGTGCGAGCTATCCGGGTGCGTATCCGGGTCAGGCTCCGCCGGGTGCGTACCCGGGCCAAGCTCCGCCGGGTGCTTATCCTGGTGCGCCGGGCGCGTACCCGGGTGCGCCGGCGCCGGGCGTGTACCCGGGTCCGCCGAGCGGTCCGGGCGCGTATCCGAGCAGCGGCCAGCCGAGCGCGCCGGGTGCGTATCCGGCGACCGGCCCGTATGGTGCGCCGGCGGGTCCGCTGATTGTTCCGTATAACCTGCCGCTGCCGGGTGGCGTGGTTCCGCGTATGCTGATCACCATTCTGGGCACCGTGAAGCCGAACGCGAACCGTATCGCGCTGGACTTCCAACGTGGTAACGATGTTGCGTTCCACTTTAACCCGCGTTTTAACGAGAACAACCGTCGTGTGATTGTTTGCAACACCAAACTGGACAACAACTGGGGCCGTGAGGAACGTCAGAGCGTGTTCCCGTTTGAGAGCGGCAAGCCGTTCAAAATTCAAGTGCTGGTTGAACCGGACCACTTTAAGGTGGCGGTTAACGATGCGCACCTGCTGCAGTACAACCACCGTGTTAAGAAACTGAACGAAATCAGCAAACTGGGCATCAGCGGTGACATTGATCTGACCAGCGCGAGCTATAACATGATTCTCGAG, with restriction sites underlined.

### Amino acid sequence

MAHAMENSWTISKEYHIDEEVGFALPNPQENLPDFYNDWMFIAKHLPDLIESGQLRERVEKLNMLSIDHLTDHKSQRLARLVLGCITMAYVWGKGHGDVRKVLPRNIAVPYCQLSKKLELPPILVYADCVLANWKKKDPNKPLTYENMDVLFSFRDGDCSKGFFLVSLLVEIAAASAIKVIPTVFKAMQMQERDTLLKALLEIASCLEKALQVFHQIHDHVNPKAFFSVLRIYLSGWKGNPQLSDGLVYEGFWEDPKEFAGGSAGQSSVFQCFDVLLGIQQTAGGGHAAQFLQDMRRYMPPAHRNFLCSLESNPSVREFVLSKGDAGLREAYDACVKALVSLRSYHLQIVTKYILIPASQQPKENKTSEDPSKLEAKGTGGTDLMNFLKTVRSTTEKSLLKEGGSGGGSGGSGGSGGEFADNFSLHDALSGSGNPNPQGWPGAWGNQPAGAGGYPGASYPGAYPGQAPPGAYPGQAPPGAYPGAPGAYPGAPAPGVYPGPPSGPGAYPSSGQPSAPGAYPATGPYGAPAGPLIVPYNLPLPGGVVPRMLITILGTVKPNANRIALDFQRGNDVAFHFNPRFNENNRRVIVCNTKLDNNWGREERQSVFPFESGKPFKIQVLVEPDHFKVAVNDAHLLQYNHRVKKLNEISKLGISGDIDLTSASYNMILEHHHHHH

### LPS administration at the hock as a model of inflammation

B6 mice were administered 2.1 μg IDO-Gal3 in 40 μl PBS subcutaneously (ipsilateral and contralateral) at the region of the hock and challenged with 2 ng g^−1^ lipopolysaccharide (LPS) in 40 μl, after 24 h or 120 h post IDO-Gal3 treatment, to assess local and distal modulation of inflammation. At 2 h after LPS administration, animals were euthanized and the injection site collected.

### Quantitative PCR

Soft tissue was separated from bone and stored in RNAlater RNA stabilization reagent (Qiagen) in preparation for qPCR. Soft tissues were homogenized and RNA purified using RNeasy Protect mini kit (Qiagen). Complementary DNA was synthesized from RNA using the High-Capacity cDNA Reverse Transcriptase kit (ThermoFisher) for use in qPCR in accordance with manufacturer instructions. qPCR analysis was run with primers specific for pro-inflammatory cytokines (IL-12a, IL-12b, IL-1*β*, IFN-*γ* and IL-6) using Applied Biosystems QuantStudio 12K Flex Real-Time PCR System. Results are presented as the ratio of gene expression to glyceraldehyde 3-phosphate dehydrogenase (GAPDH) expression determined by the relative quantification method. Treatment groups were normalized to PBS only group.

### Housing conditions

Mice were housed in individually ventilated cages with corn cob bedding, provided reverse osmosis water and standard diet, 14 h:10 h dark:light cycle, 68–79 °F room temperature and 30–70% relative humidity.

### Hock infiltration

Tissues were fixed with 10% neutral formalin (pH 7.4) overnight. After fixation, tissues were washed in deionized water and decalcified by storing in 10% ethylenediaminetetraacetic acid (EDTA) at 4 °C for 3 weeks. Samples were assessed every 2–3 d for stiffness and EDTA solution replenished. Tissues were submitted in 70% ethanol to the University of Florida Molecular Pathology Core for processing, paraffin embedding, sectioning, mounting and staining with haematoxylin and eosin (H&E). Tissues were imaged using a Zeiss Axiovert 200M with a ×20 objective lens through the multidimensional acquisition module. Eleven images were taken per tissue and scored by two blinded independent individuals on the basis of cellular infiltration (0: absent, 1: mild, 2: moderate, 3: severe) and epidermis hypertrophy (0: 0–20 μm in thickness, 1: 21–40 μm, 2: 41–60 μm, 3: 61 μm or above).

### Mass spectrometry

IDO-Gal3 (2.2 μg) in 40 μl PBS or 40 μl PBS alone was injected into the hock site of B6 mice (*n* = 3). At 30 min after injection, the mice were euthanized, the hock and tibia tissue regions excised and flash frozen using liquid nitrogen. These samples were then submitted to the Southeast Center for Integrated Metabolomics at the University of Florida for mass spectrometric analysis of kynurenine levels.

### Serum cytokine analysis

At the prescribed endpoints, animals were placed under deep terminal anaesthesia and cardiac punctures performed. Blood (2 ml) was collected in microtainer serum separator tubes and centrifuged at 1,500 r.p.m. for 5 min at room temperature. The serum was collected and stored at −20 °C. Cytokine analysis was performed using a Luminex 200 system running xPONENT 3.1 software (Luminex) following manufacturer instructions.

### Imiquimod-induced psoriasis

Pre-clinical modelling of psoriasis was carried out in 8–12-week-old female C57BL/6j mice (Jackson Laboratory) in accordance with the Institutional Animal Care and Use Committee (IACUC) at the University of Florida. The model used was a modified version of a previously reported model^37^. Briefly, mice were anaesthetized and their backs were shaved, followed by application of depilatory cream to remove any remaining fur. Each day for 14 d total, 5% IMQ cream (62.5 mg, Patterson Veterinary Supply, 07–893-7787) was applied to the backs of the mice. On the 3rd day of IMQ application, mice were subcutaneously injected with five 10 μg doses of IDO-Gal3 in sterile saline distributed evenly throughout the back, a molar equivalent of NL-Gal3 delivered in the same volume, or a sterile saline control (*n* = 12 per group). Disease severity was measured each day using a modified version of the Psoriasis Area and Severity Index (PASI) where area of effect is not taken into account. Erythema (redness), scaling and thickening were scored independently and assigned a score on a scale of 0 to 4: 0, none, 1: slight, 2: moderate, 3: marked, 4: very marked. The cumulative score was reported as a measure of the severity of inflammation (scale 0–12).

### Near-infrared conjugation and in vivo imaging of IDO-Gal3 injected subcutaneously

In vivo imaging of fluorescently tagged IDO-Gal3 was carried out in 8–12-week-old female C57BL/6j mice (Jackson Laboratory) in accordance with the IACUC at the University of Florida. Before injection, IDO-Gal3 was incubated with IRDye 680RD NHS Ester (LI-COR, 929–70050) following manufacturer instructions. Mice received a 40 μl (2.1 μg, 3.27 μM) subcutaneous injection of fluorescently labelled IDO-Gal3 to the hock. Immediately following injection and every subsequent 24 h following injection, mice were imaged using an IVIS Spectrum in vivo imaging system (PerkinElmer). Fluorescent images were captured using the AF680 emission filter, subject size 1.5 cm, 0.2 s exposure time, field of view B (6.6 cm), medium binning (factor of 8) resolution and a 1F/Stop aperture. Relative fluorescent intensities were represented by a pseudo colour scale ranging from red (least intense) to yellow (most intense).

### In vivo imaging of tissue distribution after hock injection

NanoLuc-Gal3 (164 pmol) in 40 μl PBS was injected subcutaneously into the hock of B6 mice. At the prescribed time points, animals were euthanized in accordance with approved protocols. Organs and tissues of interest were collected, weighed, processed, incubated with furimazine and bioluminescence quantified by a luminometer. Bioluminescence images were acquired using an IVIS Spectrum in vivo imaging system. Living Image software v4.3.1 (PerkinElmer) was used to acquire the data immediately after furimazine administration. Exposure time for the bioluminescence imaging was 1 s. Regions of interest (ROIs) were quantified as the average radiance (photons s^−1^ cm^−2^ sr^−1^). The specific amount of protein in tissue was determined by comparison with a standard curve of NanoLuc-Gal3 activity.

### Oral *Listeria* infection after hock injection

Immune suppression from IDO-Gal3 was evaluated in response to oral infection with *Listeria monocytogenes* InIA^M^ (strain 10403s). Tissue bacteria burdens in the liver and spleen were used as a metric for infection where the liver is local for gut infection and the spleen represents systemic spread of infection. On the day before infection, 10-week-old naïve C57BL/6 mice (Taconic Biosciences) received IDO-Gal3 injection subcutaneously (hock), while control mice received sterile saline injection. The following day, mice were orally infected with 2 × 10^9^ colony-forming units (c.f.u.) of *Listeria monocytogenes* per mouse. To prepare for infection, bacteria were cultured overnight in BHI broth at 37 °C, shaking at 220 r.p.m. Before infection, a subculture of 2 ml of the bacteria and 18 ml BHI broth was cultured under the same conditions until reaching an OD_600_ = 0.8. Bacteria were pelleted, resuspended in 500 μl of sterile PBS, and 50 μl of bacteria was pipetted onto a small square of white bread and fed to each mouse individually. Once the entire piece of bread was eaten, mice were returned to their cage. At day 7 post infection, mice were euthanized, followed by collection of the spleen and liver. Tissue was homogenized, suspended in 1% saponin for 1 h, then plated at serial dilutions from undiluted to 1:1,000. BHI Agar plates were treated with streptomycin to limit non-specific bacterial growth, as this strain of *L. monocytogenes* is streptomycin resistant. C.f.u.s were counted at 24 h post-plating.

### Murine model of periodontal disease

All mice were lavaged with 25 μl 0.12% chlorhexidine gluconate (3 M) for 3 d. On days 4, 5, 6 and 7, mice received a 25 μl oral lavage with 2.5 × 10^9^ *P. gingivalis* strain 381 and 2.5 × 10^9^ *Aggregatibacter actinomycetemocomitans* strain 29522 (ATCC) resuspended in 2% low-viscosity carboxy-methyl-cellulose (Sigma-Aldrich). Oral lavage was repeated every week for 5 weeks. Each week, 1 d before the first day of infection (prophylactic) or 1 d after the last day of infection (therapeutic), 10 μl of IDO-Gal3 was injected into the submandibular space using a 30-gauge insulin syringe (Becton Dickenson). Each week of infection, on the first day of infection, before infection, microbial sampling of the oral environment was performed with calcium alginate swabs (ThermoFisher). One week following the last infection, the mandibles were collected to evaluate soft tissue soluble mediator expression and bone morphometric analysis.

### Oral soft tissue cytokine expression

Mandibles with both soft tissue and bone were subjected to bead beating at two 2 min intervals with 2 min of cooling in between using 1.0-mm-diameter zirconia silica beads (BioSpec) in cell extraction buffer (ThermoFisher). The buffer was prepared with a protease inhibitor cocktail (mini cOmplete, Roche) and PMSF protease inhibitor (Abcam) to allow for dissociation and lysis of all soft tissue while leaving the hard tissues intact. MILLIPLEX Multiplex assays (EMD Millipore) were used to probe resulting lysates for IL-6, IL-1*β*, IL-10 and MCP1 according to manufacturer protocols. Data were acquired on a Luminex 200 system running xPONENT 3.1 software (Luminex) and analysed using a 5-paramater logistic spline-curve fitting method using Milliplex Analyst v5.1 software (Vigene Tech). Data are presented as pg ml^−1^ normalized to total protein (BCA assay; ThermoFisher Pierce).

### Mandible bone morphometric analysis

Mandibles were fixed in 4% buffered formalin for 24 h, stored in 70% alcohol and scanned at 18 μm resolution using a micro-CT system (Skyscan). Three-dimensional images were reconstructed and the resulting images re-oriented spatially using anatomical landmarks with the NRecon and DataViewer software (Skyscan). A standardized 5.4 mm^3^ ROI was set with standardized dimensions of 1.5 (frontal) × 4.0 (sagittal) × 0.9 mm (transversal). Anatomical landmarks were used for the standardized positioning of the ROI: frontal plane, the roof of the furcation area between mesial and distal roots of the upper first molar; sagittal plane, anterior limit was the distal aspect of the mesial root of the first molar. The thickness of the ROI on the transversal plane was set to 50 slices (900 μm) and counted towards the palatal/medial direction beginning from the image that included the centre of the upper first molar in its transversal width. A standardized threshold was set to distinguish between non-mineralized and mineralized tissues where total volume and total thickness of the ROI were calculated. Mean trabecular bone volume (BV) and thickness (BT) analysis assessed the percentage of mineralized tissue (BV; BT) within the total volume/thickness (TV; TT) of the ROI and is presented as the BV/TV ratio (mean trabecular bone volume) or BT/TT ratio (mean trabecular thickness). Vertical bone loss is the distance from the cementoenamel junction (CEJ) to the alveolar bone and was calculated at 12 sites over three molars and averaged to calculate the average vertical bone loss in mm.

### Oral bacterial burden

Genomic DNA was isolated from microbial sampling of the oral environment using a DNeasy kit (Qiagen) according to manufacturer instructions. The gDNA was then probed for *P. gingivalis* 16S, *A. actinomycetemocomitans* 16S and total 16S using real-time PCR. The percentage of *A. actinomycetemocomitans* 16S and *P. gingivalis* 16S within the total 16S compartment was calculated using the following formula: Ct value of total 16S/Ct value of *A. actinomycetemocomitans* or *P. gingivalis* 16S. 16s rRNA For: AGA GTT TGA TCC TGG CTC AG; Rev: ACG GCT ACC TTG TTA CGA CTT; Pg For: CTT GAC TTC AGT GGC GGC AG; Rev: AGG GAA GAC GGT TTT CAC CA; Aa For: GTT TAG CCC TGG CCG AAG; Rev: TGA CGG GCG GTG TGT ACA AGG.

### Mechanical overloading osteoarthritis murine model

IDO-Gal3 activity was assessed in a post-traumatic osteoarthritis mouse model^30^ that applies cyclic mechanical loading to the knees of aged (6 months) mice, causing mechanical damage and consequent inflammation and cartilage degradation^30,31^. The mice were anaesthetized and placed in a fixture with the knee in flexion; loading (9 N) was applied axially for 500 cycles and loading sessions were done on the mice 5 times per week during the experiment. IDO or IDO-Gal3 was prepared at 143 μM, and 20 μl of each treatment was injected intra-articularly at the start of each week of the study, with each knee receiving 4 total treatments. At the end of the 4-week study, mice were euthanized for analysis of gene expression and histopathology.

### Mechanical overloading osteoarthritis pharmacokinetics analysis

Pharmacokinetics of IDO and IDO-Gal3 retention after local injection at the disease site was assessed by intravital imaging over the course of 7 d. Both proteins were labelled with Li-Cor IRDye 680RD NHS ester (Li-Cor Biosciences) to visualize and measure protein knee retention. Mice were subjected to mechanical loading for 2 weeks before each treatment was administered via intra-articular injection. Intravital imaging was performed (excitation 672 nm, emission 694 nm) immediately following injection and every subsequent 24 h. The fluorescence signal measured at the joint over time was normalized to the initial measurement for each knee and an exponential decay was individually fit for each specimen. The AUC/bioavailability for each joint was calculated from the best-fit line of exponential decay. After 7 d, the mice were euthanized and an ex vivo image was taken of each joint with the surrounding skin removed to increase measurement sensitivity.

### Mechanical overloading osteoarthritis, gene expression analysis

Gene expression was evaluated by *Taq*Man qPCR in the knee joint and the popliteal lymph node that drains the knee joint. Following euthanization, knees and popliteal lymph nodes were excised. Combined joint tissue from the synovial wall and articular surface (not exceeding 30 mg total) and the popliteal lymph nodes were homogenized with bead pulverization in Qiazol. RNA was extracted and purified using the RNeasy Plus mini kit from Qiagen and quantified using NanoQuant plate from Tecan in a microplate reader (Tecan Infinite 500, Tecan). The RNA was converted to cDNA using the iScript Synthesis kit from Bio-Rad (Hercules). Gene expression was calculated by the ΔΔCt method, normalizing to GAPDH and beta-actin (ACTB). *Taq*Man reagents were purchased from ThermoFisher and used according to provided protocols, using appropriate primers (IL-12*β*: Mm01288989_m1, IL-6: Mm01210732_g1, MMP13: Mm00439491_m1, TNF-*α*: Mm00443258_m1, GAPDH: Mm99999915_g1, ACTB: Mm02619580_g1).

### Mechanical overloading osteoarthritis, histologic staining and scoring

Tissue samples were fixed in 10% formalin and decalcified in 20% EDTA for 7 d. A standard 8 h cycle of graded alcohols, xylenes and paraffin wax was used to process tissues before embedding and sectioning at 5 μm thickness. Sections were mounted on positively charged glass slides and stained with H&E using the Gemini autostainer (ThermoFisher). Safranin O staining was performed using the StatLab staining kit. Sections were imaged using Leica SCN400 slide scanner. Each joint was evaluated by at least two mid-frontal sections for both H&E and safranin O stains. A board-certified veterinary pathologist conducted histopathologic interpretations under blinded conditions^38^. OARSI scoring was based on medial and lateral tibial plateaus (scale of 0–6)^39^, and a generic score was concurrently assigned on the basis of H&E features and the safranin O staining of the tibial plateaus according to the DJD methodology (DJD severity, scale 0–3).

### Surgically induced osteoarthritis, rat model

Sixteen male Lewis rats (12–14-week-old, approximately 250 g) were acquired from Charles River Laboratories. Rats were acclimated to the University of Florida housing facilities for 1 week. After acclimation, rats underwent baseline gait and von Frey behavioural testing. Following baseline behavioural testing, all rats received medial collateral ligament plus medial meniscus transection (MCLT + MMT) surgery to their right hind limb. Gait data were collected on weeks 3, 5 and 7 post-surgery using a Phantom Miro Lab320 (Phantom Camera Control 3.0), while von Frey testing was conducted weekly after surgery. At 8 weeks post-surgery, rats received a unilateral saline or IDO-Gal3 injection. Rats underwent von Frey testing the day following injection and gait testing 2 d after injection. Both gait and von Frey data were collected weekly until euthanasia.

### Surgically induced osteoarthritis, behavioural study

MCLT + MMT surgery rats were anaesthetized in a 2.5% isoflurane (Patterson Veterinary) sleep box. Rats were then aseptically prepped with betadine surgical scrub (Purdue Products) and 70% ethanol in triplicate, and transferred to a sterile field with anaesthesia maintained via mask inhalation of 2.5% isoflurane. During MCLT + MMT surgery, a 1–2 cm midline skin incision was made along the medial aspects of the rat knee and the skin was retracted to reveal the medial collateral ligament. The medial collateral ligament was transected and the knee was placed in the valgus orientation to stretch the medial compartment and expose the medial meniscus. The medial meniscus was cut radially, and absorbable 5–0 vicryl braided sutures (Ethicon) were used for muscle closure and 4–0 ethilon nylon monofilament sutures (Ethicon) were used for skin closure. Rats recovered post-operatively in a warming box until weight bearing on all limbs. For pain management, rats received a subcutaneous injection of buprenorphine (0.05 mg kg^−1^) (Patterson Veterinary) intra-operatively and every 12 h for 48 h. Rats were grouped into a saline injection cohort (*n* = 7) or IDO-Gal3 injection cohort (*n* = 8). At 8 weeks after OA induction, rats received 30 μl unilateral injections of either sterile saline or IDO-Gal3 (33 pg μl^−1^) in the operated knee using sterile allergy syringes (1 ml, 27G × 3/8) (Becton Dickinson). First, rats were anaesthetized using 2.5% isoflurane (Patterson Veterinary) and the operated knee was aseptically prepped as described above. Then the needle was inserted through the patellar ligament following the patellar groove into the joint space. The knee was flexed and the injection site was cleaned with sterile gauze and 70% ethanol.

### Surgically induced osteoarthritis, tactile sensitivity

Tactile sensitivity was assessed by measuring the 50% paw withdrawal threshold determined using the Chaplan up-down method for von Frey filaments^1^. Before surgery, animals were acclimated to the wire mesh-floored cage. Rats underwent von Frey testing the week before surgery and weekly after surgery for 12 weeks. On week 8, rats were injected, then underwent von Frey testing the following day. During von Frey testing, a von Frey filament series (0.6, 1.4, 2.0, 4.0, 6.0, 8.0, 15.0 and 26.0 g) was applied to the plantar region of each hind foot. First, the 4.0 g von Frey filament was applied. A less-stiff filament was applied if a paw withdrawal occurred, and a stiffer filament was applied if a paw withdrawal did not occur. Using these data, the force at which rats were equally likely to withdraw or tolerate was calculated via Chaplan’s approximation^1^.

### Surgically induced osteoarthritis, magnetic capture

Commercially available streptavidin-functionalized particles (Dynabeads MyOne Streptavidin C1, 65001; Life Technologies) were coated with biotinylated antibodies for CTXII (AC-08F1, ImmunoDiagnostic Systems), IL-6 and MCP1 (517703 and 505908, Biolegend). Here, particles were washed 3 times in PBS, incubated for 2 h on a tube revolver at room temperature in antibody mixes that contained either 8.9 ng μl^−1^ anti-CTXII, 8.9 ng μl^−1^ anti-IL-6 or 357 ng μl^−1^ anti-MCP1 and then moved to static incubation at 4 °C overnight. Particles were then washed three times in PBS containing 2% BSA and 2 mM EDTA (capture buffer), with final antibody amounts per particle measured to be 0.33 ng Ab μg^−1^ particle (anti-CTXII), 0.33 ng Ab μg^−1^ particle (anti-IL-6) and 13.0 ng Ab μg^−1^ particle (anti-MCP1).

Following euthanasia via exsanguination, magnetic capture was performed in both the operated and contralateral knee, as previously described^40^. Briefly, 300 μg of antibody-conjugated magnetic particles (equal parts for each particle type) were suspended in 10 μl of saline, then injected in the operated and contralateral knee. After 2 h incubation in the knee, particles were collected via 5 repeated 50 μl PBS washes of the knee, with collected fluid combined and particles in the fluid isolated via a magnetic separator. Collected particles were washed twice with capture buffer, then incubated for 15 min in the 100 mM Glycine-Tris buffer (pH 3.1) containing 2% BSA and 2 mM EDTA (release buffer). Following biomarker release, magnetic particles were isolated by magnetic separation and the pH of the supernatant was adjusted to 8.3 for enzyme-linked immunosorbent assays (ELISA). CTXII was then measured in the supernatant using the Cartilaps CTXII ELISA kit (AC-08F1, ImmunoDiagnostic Systems Cartilaps kit) according to manufacturer instructions, and CCL2 was quantified using a rat CCL2 ELISA kit (KRC1012 Life Technologies) according to manufacturer instructions and previously described modifications^41^. IL-6 was quantified using ELISA developed in the laboratory using biotin anti-rat IL-6 antibody (517703) and purified (coating) anti-rat IL-6 antibody (517701, Biolegend). Coating antibody was diluted in 100 mM NaHCO_3_ and 34 mM Na_2_CO_3_ (pH 9.5), placed in coated microwells (Nunc MaxiSorp, 434797; ThermoFisher), incubated for 30 min on a plate shaker then overnight at 4 °C, washed 5 times with PBS with 0.5% Tween-20 (wash buffer), blocked for 1 h with PBS containing 2% BSA and 10% heat-treated bovine serum, and finally washed 5 times with wash buffer. Samples and standards were pre-incubated with anti-IL-6 detection antibody (30 min at room temperature and then overnight at 4 °C), then added to ELISA plate and incubated for 3 h. The plate was then washed 5 times, and 100 μl of avidin-HRP (405103, Biolegend, diluted 500 times in 2% BSA and 10% heat-treated bovine serum) was added to the plate and incubated for 30 min. The plate was again washed 5 times, with 100 μl of tetramethylbenzidine substrate added for 15 min, followed by 100 μl of stop solution (diluted sulfuric acid). Absorbance was read at 450 and 650 nm. Particles were quantified in 60 μl of capture buffer, with particle suspensions read for absorbance at 450 nm using Synergy 2 Multi-Mode microplate reader, as previously described^40^.

### Surgically induced osteoarthritis histology

Following magnetic capture, operated and contralateral knees were dissected and placed in 10% neutral buffered formalin (ThermoFisher) for 48 h at room temperature. Following fixation, knees were decalcified using Cal-Ex (ThermoFisher) for 5 d at room temperature, dehydrated through an ethanol ladder and embedded in paraffin wax via vacuum infiltration. Then, 10 μm frontal sections were acquired, with at least one section taken at every 100 μm through the loading region of the medial meniscus. Slides were stained with toluidine blue.

### Surgically induced osteoarthritis-joint-retention study

Sixteen male Lewis rats (12–14-week-old, approximately 250 g) were acquired from Charles River Laboratories. Rats were acclimated to the University of Florida housing facilities for 1 week. Then rats underwent MCLT + MMT surgery. After 8 weeks, rats received a 50 μl injection of either Nano-Glo Luciferase (NL) or Nano-Glo Luciferase Galectin-3 (NL-Gal3). Rats were IVIS imaged immediately following injection, then at 1, 2, 4, 8, 12, 16, 20, 24 and 28 d post-injection. After imaging on day 28, rats were euthanized, and both operated and contralateral knees were dissected for joint tissue distribution analysis via IVIS. In vivo joint retention was measured in the operated and contralateral knees of 16 male Lewis rats. At 8 weeks post MCLT + MMT surgery, knees were aseptically prepped and rats received bilateral injections of NL (50 μl, 3.27 μM) (*n* = 8) or bilateral injections of NL-Gal3 (50 μl, 3.27 μM) (*n* = 8) in both operated and contralateral knees. (Surgeries and injections were conducted the same way as described above). Knees were flexed, then injected with 50 μl furimazine (50 μl, 1:50 dilution in PBS). Immediately after injection, knees were flexed again and luminescence was measured with IVIS using a 1 s (and 60 s) exposure time in field of view D. Furimazine injections and IVIS imaging were repeated at 1, 2, 4, 8, 12, 16, 20, 24 and 28 d post NL or NL-Gal3 injection. For analysis, a ROI was drawn around the largest luminescent signal and copied to create an identical sized ROI for all knees.

After imaging on day 28, rats were euthanized via cardiac puncture under deep isoflurane anaesthesia. Knees were dissected to isolate the patellar tissue, tibial tissue, femoral tissue and meniscus. The patellar tissue included the patella, patellar synovium and fat pad. The tibial tissue included the tibia and attached synovium. The femoral tissue included the femur and attached synovium. Tissues were placed in 24-well plates and incubated with furimazine (1:50 dilution in PBS) at room temperate for roughly 1 min. After incubation, tissues were removed from the 24-well plates and luminescence was measured with IVIS using auto-exposure in field of view C. For assessment, individual ROIs were drawn around the patellar tissue, tibial tissue, femoral tissue and meniscus. The ROI for each tissue was copied to create an identical sized ROI for the respective tissue.

### Statistical analysis

Statistical analyses were performed using GraphPad Prism 8 and 9, with the following exceptions. Surgically induced osteoarthritis data were analysed using R Analytics 4.0.4 with RStudio 2022, and MATLAB 2020b was used for Mann-Whitney *U*-tests on psoriasis data. Study-specific analyses are reported in figure captions.

### Reporting summary

Further information on research design is available in the Nature Portfolio Reporting Summary linked to this article.

### Data availability

The main data supporting the results in this study are available within the paper and its Supplementary Information. The source datasets are available for research purposes from the corresponding authors on reasonable request.

### Code availability

The codes for gait-data analysis are available at https://github.com/OrthoBME, https://github.com/OrthoBME/GAITORsuite and https://github.com/OrthoBME/GEKO.

## Supplementary Material

Supplementary Information
