# Supplementary material for: Suppression of local inflammation via galectin-anchored indoleamine 2,3-dioxygenase
Source: Nat Biomed Eng. Author manuscript; Available in PMC 2023 Sep 27. (PMC10504068; doi:10.1038/s41551-023-01025-1)
Supplement: Supplementary Information [file NIHMS1904343-supplement-Supplementary_Information.pdf]

nature biomedical engineering

Article https://doi.org/10.1038/s41551-023-01025-1

Suppression of local inflammation via galectin-anchored indoleamine 2,3-dioxygenase

In the format provided by the authors and unedited

Supplementary methods

Immunization

To study the generation of antibodies, female C57BL/6J mice of 8 - 10 weeks old were purchased from Jackson Laboratories. Cohorts of female mice (n = 5) received a 100 μL scruff injections at day 0 of the following formulations: 10 μg of IDO-Gal3, 2 μg of IDO-Gal3, or 5 μg of IDO-Gal3 emulsified in TiterMax. Mice cohort that received 2 μg of IDO-Gal3, received a 100 μL injection challenge with 2 μg of IDO-Gal3 at day 7, 14, 21 and 28. Mice cohort that received 5 μg of IDO-Gal3 with adjuvant, received a 100 μL injection with 5 μg of IDO-Gal3 emulsified in TiterMax at day 21. Blood was drawn at day -1, day 7, day 21, and day 35 from the facial vein.

Method for total IgG ELISA

Sera was analyzed for anti-IDO-Gal3 total IgG antibodies via ELISA by adapting established methods. Briefly, the plates were coated with saline buffer or 1 μg/mL IDO-Gal3 and incubated overnight at 4 °C. Plates were washed three times with 0.5% Tween-20 in PBS (PBST) and blocked with 150 μL of 1% bovine serum albumin (BSA) in PBST for 1 h at room temperature. Serum was diluted (dilution factor: 1:100, 1:1000, 1:10000) with 1x PBS having 1% BSA, added to the blocked wells (100 μL), and incubated for 1 h at room temperature. Serum was removed and plates were washed three times with PBST. 100 μL of peroxidase-conjugated goat anti-mouse IgG was added to each well (1:5000 in PBS with 1% BSA) and incubated for 1 h at room temperature. Secondary antibody solution was removed, and plates were washed five times with PBST. Plates were developed with 100 μL of TMB substrate for 5 min at room temperature. Finally, 100 μL of stop solution (0.16 M sulfuric acid) was added, and absorbance was measured at 450 nm using a SpectraMax M3 plate reader.

Data analysis

Sera was analyzed at day -1, day 7, day 21, and day 35 using ELISA. Using the absorbance measurements from day -1, the average absorbance and standard deviation for each dilution in each treatment group was calculated. To determine positive titers, the absorbance measurement, for each dilution in each treatment group, needs to be greater than the absorbance (μ) plus 5 times the standard deviation (σ) $(\mu + 5^*\sigma)_{day-1}$, of each dilution for each treatment group. Titer 2 represents mice with absorbance measurement greater than $(\mu + 5^*\sigma)_{day-1}$ for a 1:100 dilution. Titer 3 represents mice with absorbance measurement greater than $(\mu + 5^*\sigma)_{day-1}$ for a 1:1000 dilution. Titer 4 represents mice with absorbance measurement greater than $(\mu + 5^*\sigma)_{day-1}$ for a 1:10000 dilution.

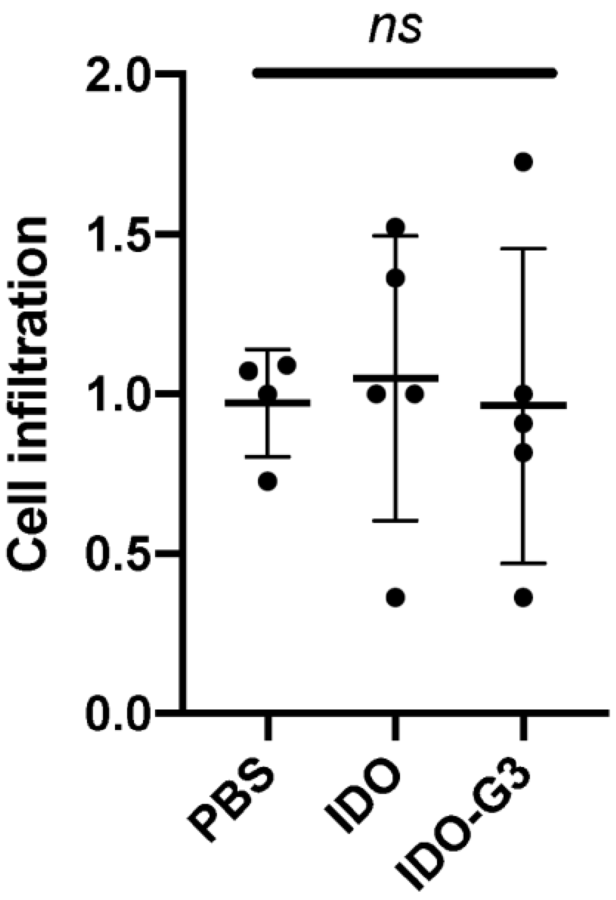

Supplementary Fig. 1 l Quantitative analysis of H&E histology images showing that subcutaneous hock injection of IDO or IDO-Gal3 alone, at 26 h and without LPS challenge (Fig. 1e), do not increase cell infiltration compared to vehicle injection alone. Data shown as mean ± s.e.m, $n = 4$, "ns" indicates $p > 0.05$, one-way ANOVA with Bonferroni's multiple comparison test. $P = 0.94$, $F = 0.06498$.

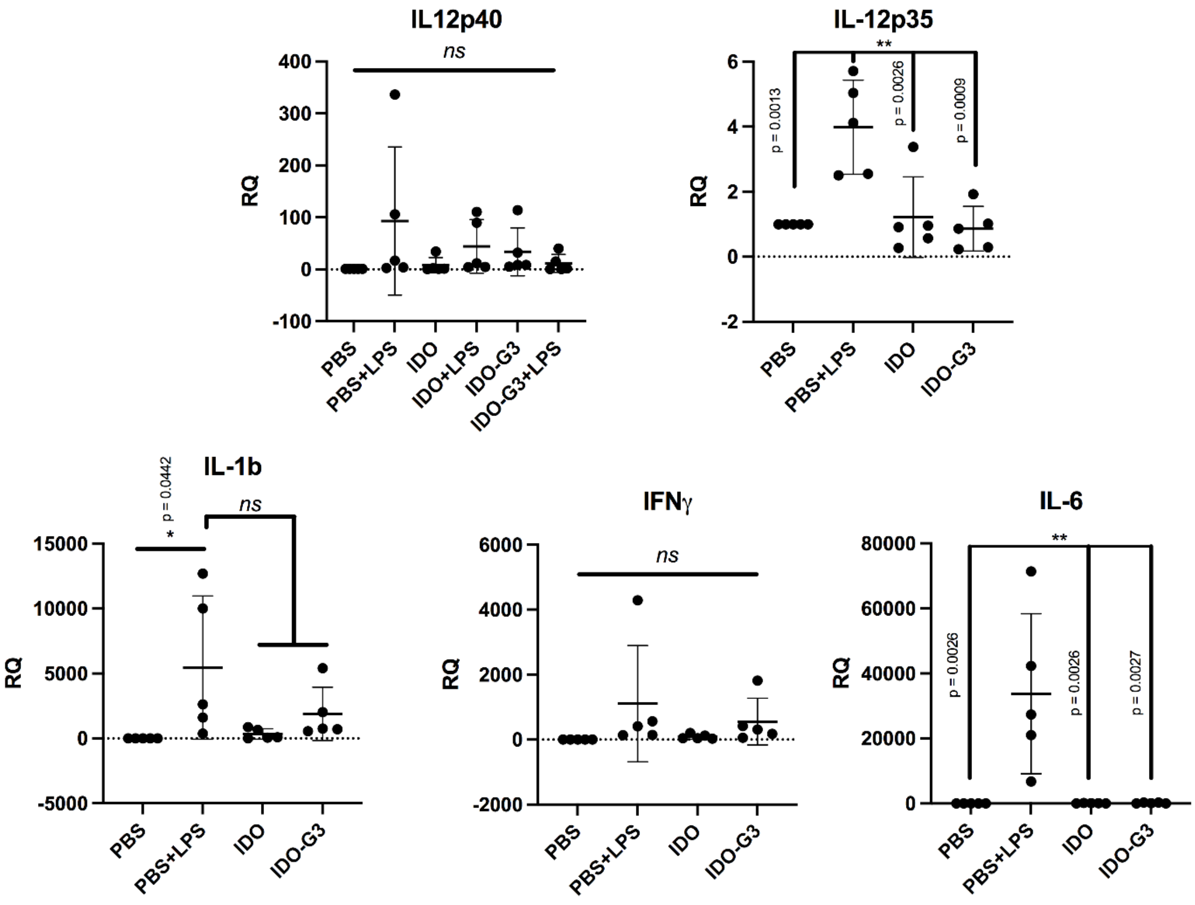

Supplementary Fig. 2 l Cytokine gene expression following subcutaneous injection of vehicle (PBS), IDO, or IDO-Gal3 at t = 0 and then vehicle or LPS (labeled as "+LPS") at t = 24. Data shown as mean ± s.e.m, n = 5, * represents $p < 0.05$, ** represents $p < 0.01$ compared to all other groups, unless otherwise indicated, one-way ANOVA with Tukey's post-hoc. F(IL12p40) = 1.371; F(IL-12p35) = 10.79; F(IL-1β) = 0.0373, F(IFN-γ) = 1.390, F(IL-6) = 6.2.

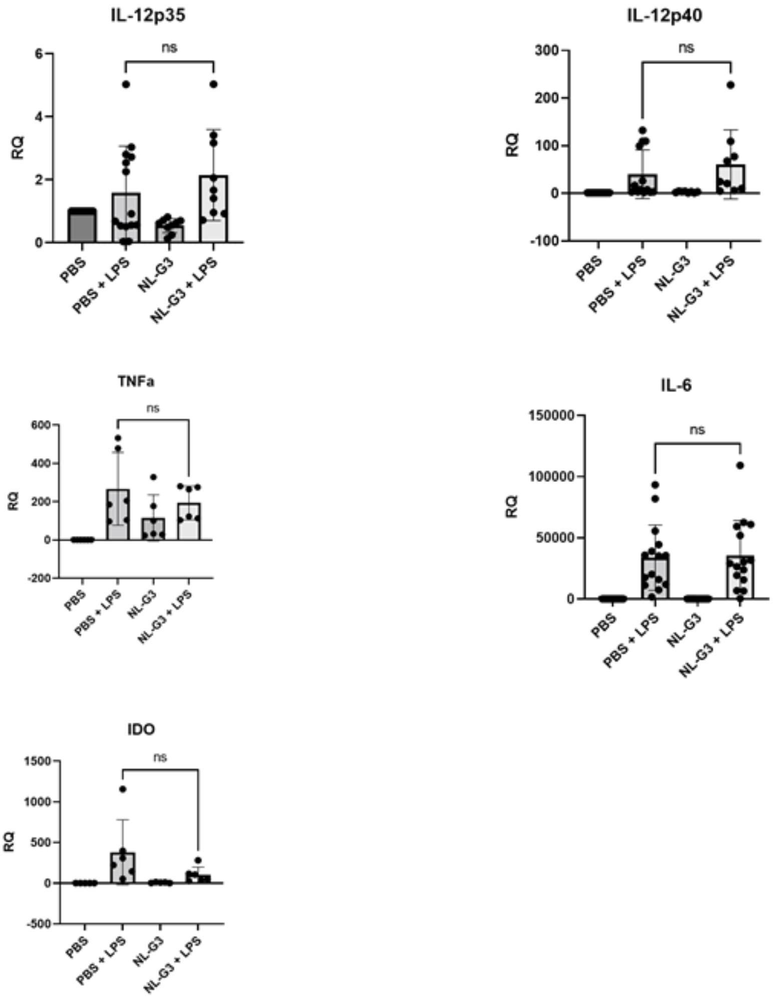

Supplementary Fig. 3 | Pretreatment with control protein NanoLuciferase –galectin 3 fusion (NL-Gal3) did not suppress inflammation in response to LPS. The LPS challenge model followed **Fig. 1e** schematic. NanoLuc-Gal3 did not suppress transcript upregulation of inflammatory cytokines IL-12p35, IL-12p40, TNF-a, and IL-6 in response to LPS challenge. Transcript levels for endogenous IDO were similarly unaffected. Data presented as mean ± s.e.m. One-way ANOVA with Tukey's post-hoc. "ns" indicates no difference; n = 6-12.

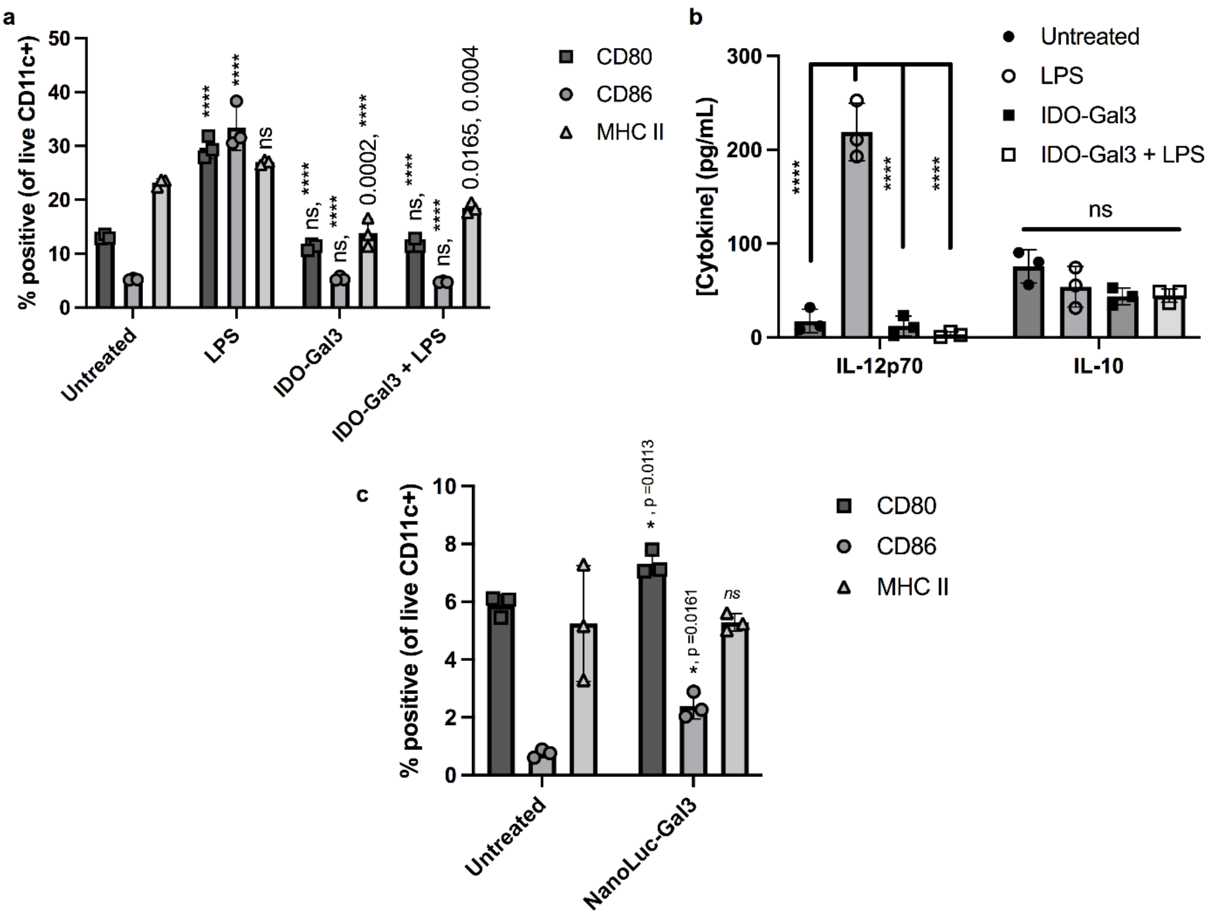

Supplementary Fig. 4 l Dendritic cells treated with IDO-Gal3 maintained an immature phenotype and resist LPS activation. Murine bone marrow-derived dendritic cells were treated with IDO-Gal3 for 24 h, washed and challenged with LPS overnight. (a) Cells were co-stained for viability, CD11c and activation markers: CD80, CD86 and MHC II, and analyzed through flow cytometry. IDO-Gal3-treated dendritic cells resist LPS-induced activation. Values shown as percent positive. (b) Supernatants were collected and analyzed for the secretion of IL-12p70 and IL-10 through ELISA. Dendritic cells treated with IDO-Gal3 do not release IL-12p70 even when stimulated with LPS, and maintain a basal IL-10 secretion. (c) Dendritic cells treated 24 h with control protein NanoLuciferase-galectin 3 (NL-Gal3) maintained immature dendritic cell phenotype, indicating the Gal3 fusion protein did not act as a damage associated molecular pattern. Replicate number, n =3; show is mean ± SEM with pair-wise significant difference from all other groups by one-way ANOVA with Tukey's post-hoc. For (a), p values reported as "X, Y", where X = p value relative to untreated and Y = p value relative to LPS positive control.

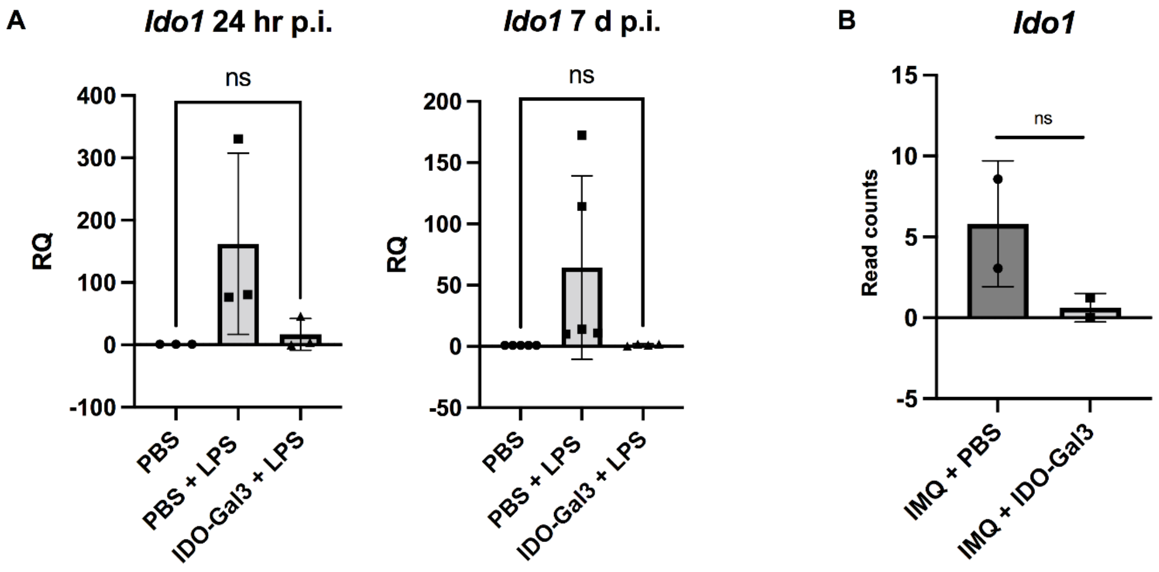

Supplementary Fig. 5 | IDO-Gal3 treatment did not induce endogenous *Ido1* expression after either LPS or imiquimod challenge. (a) Following the schedule in **Fig. 1e**, mouse hock was subcutaneously injected with either PBS vehicle or IDO-Gal3 for either 24 h or 7 d prior to 2 h subcutaneous LPS challenge. Gene expression from harvested tissue was quantified via quantitative PCR, expressed as relative quantification (RQ). Data shown as mean ± s.d; n = 3/group at 24 h post-injection; n = 5/group at 7 d post-injection (p.i.), ordinary one-way ANOVA with Tukey's post-hoc, "ns" indicates no difference between groups (24 hr: F = 3.275, p = 0.1093; 7 d: F = 3.147, p = 0.083). (b) Following the schedule in **Fig. 1l**, bulk RNA-seq was performed on skin from the psoriasis-affected area harvested at 14 d, expressed as read counts. n = 2/group, unpaired Student's t-test, "ns" indicates no difference between groups (p = 0.2075, t = 1.838, df = 2).

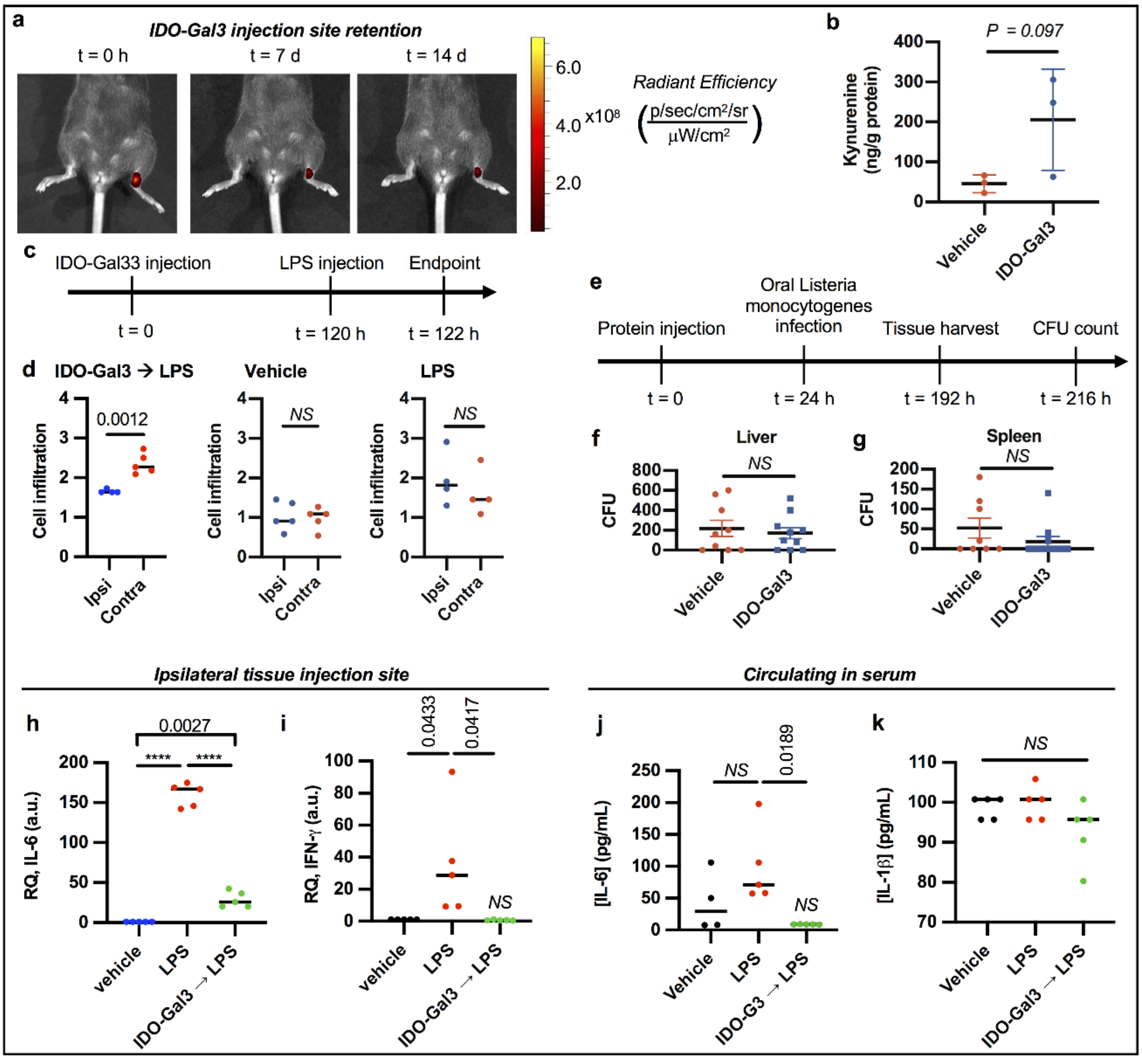

Supplementary Fig. 6 | IDO-Gal3 functions locally and is not systemically suppressive. (a) In vivo imaging of fluorophore-labeled IDO-Gal3 injected subcutaneously (s.c.) in the hock demonstrating tissue retention out to 14 d. (b) Mass spectrometry analysis quantification of kynurenine levels in hock and surrounding tissue indicating in vivo IDO enzymatic activity. (c-d) LPS challenge 120 h after s.c. hock injection at both ipsilateral (IDO-Gal3 pre-treated) and contralateral (vehicle) sites yields a local ipsilateral reduction in inflammatory cell infiltration while maintaining a robust contralateral inflammatory response. (e-g) Administering IDO-Gal3 into the s.c. hock did not alter clearance of Listeria monocytogenes after oral infection challenge, in the liver or spleen compared to vehicle control. Hock s.c. IDO-Gal3 pretreatment 120 h (c) suppre~sed transcript levels of IL-6 (h) and IL-1□ (i) at the ipsilateral LPS challenge site, blocking systemic increase in IL-6 protein (j) and maintaining baseline systemic IL-1□ (k), quantified in serum by mass spectrometry. Data presented as mean ± s.d. in (b), mean ± s.e.m. in (f-g). Statistical analyses: (b) two-tailed Student's t-test, n = 3; (d) two-tailed Student's t-test (for IDO-Gal3 > LPS, t = 5.256, df = 7; for vehicle, t = 0.3061, df = 8; for LPS, t = 0.7737, df = 6), n = 5; (f-g) two-tailed Student's t-test with Welch's correction, "NS" indicates no difference ($p > 0.05$) (for (f), t = 0.4647, df = 14.49; for (g), t = 1.220, df = 10.61); (h-k) One-way ANOVA with Tukey's post-hoc. "NS" indicates no difference and four symbols denotes $p < 0.0001$, n = 5. For (h), F = 339.4. For (i), F = 75.25. For (j), one symbol denotes p = 0.0229, F = 5.426. For (k), $p > 0.05$, F = 2.604.

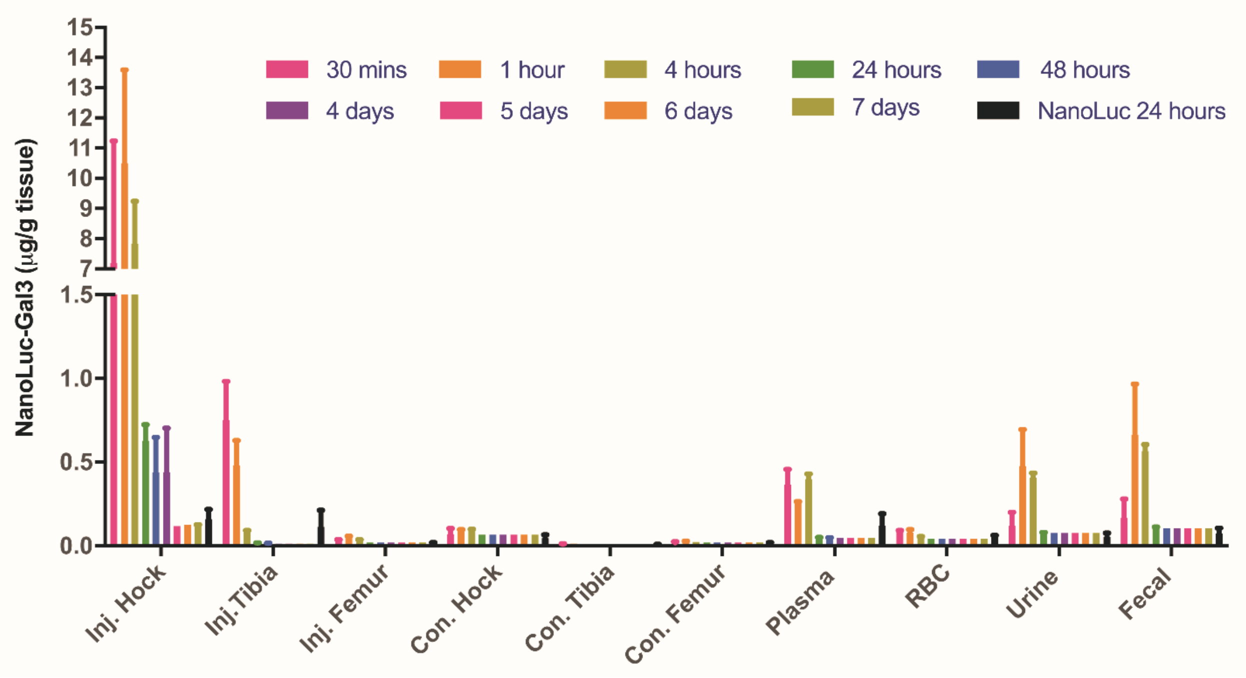

Supplementary Fig. 7 l Biodistribution of NL-Gal3 determined from NanoLuc bioluminescence at various time points following subcutaneous injection into the hock at t = 0.

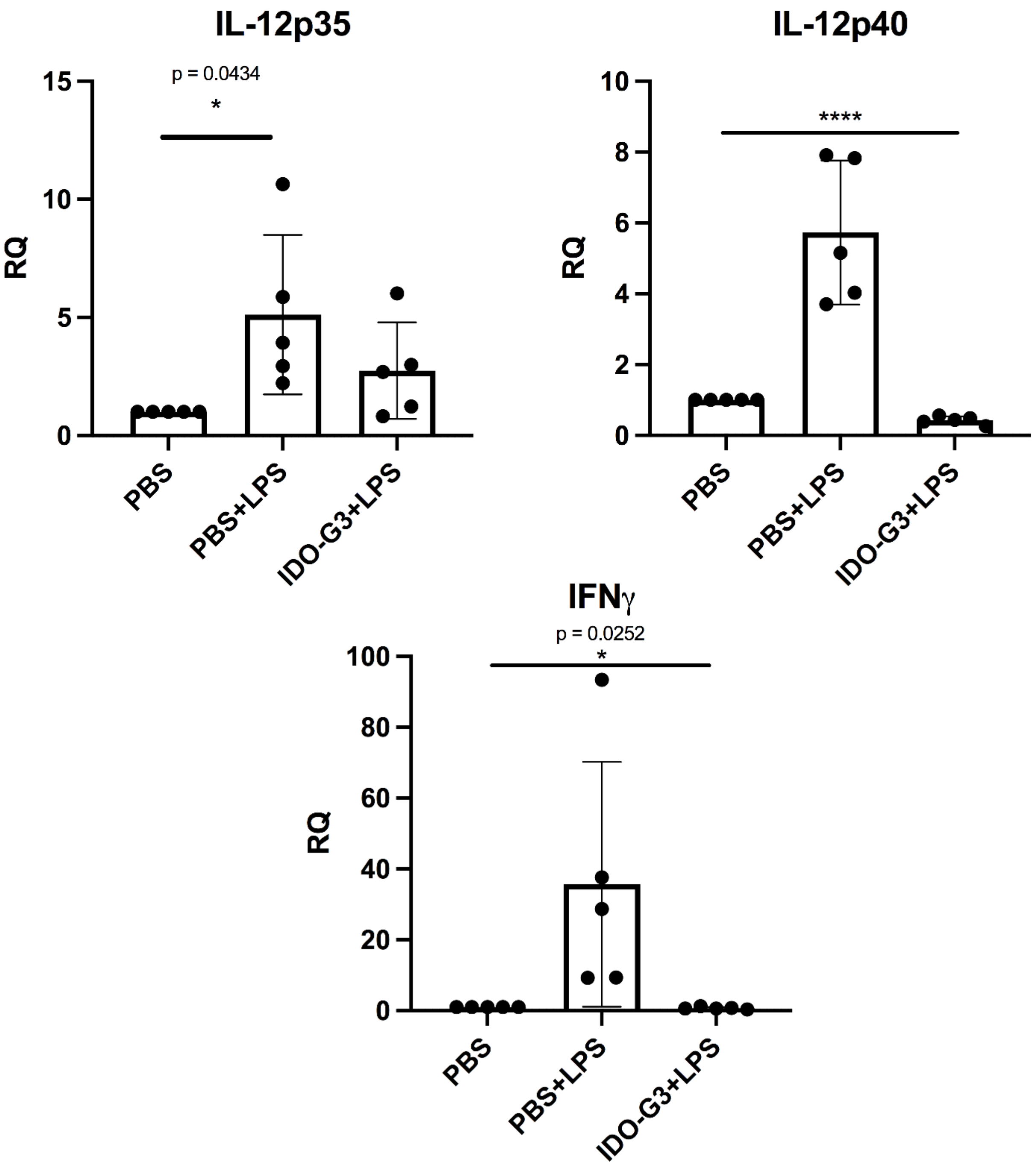

Supplementary Fig. 8 l Cytokine gene expression after subcutaneous injection of vehicle (PBS) or IDO-Gal3 at t = 0 followed by ipsilateral injection of vehicle or LPS at t = 120 h. Data presented as mean ± s.e.m., n = 5, * represents $p < 0.05$, **** represents $p < 0.0001$ between all groups relative to PBS + LPS, unless otherwise specified, one-way ANOVA with Tukey's post-hoc. F(IL-12p35) = 4.122, F(IL-12p40) = 30.61, F(IFN□) = 5.080.

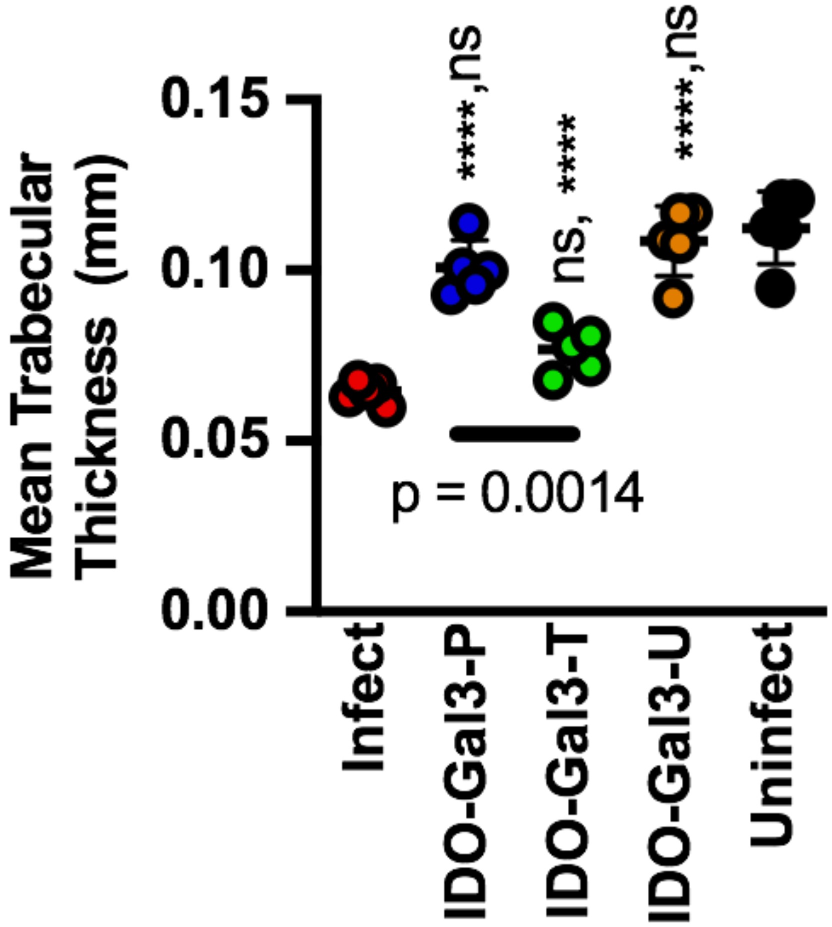

Supplementary Fig. 9 | Mean trabecular bone thickness calculated from microCT images following prophylactic (IDO-Gal3-P) or therapeutic (IDO-Gal3-T) submandibular injection of IDO-Gal3 in mice infected with PG and AA. "IDO-Gal3-U" denotes animals that received IDO-Gal3 but were not infected, "Infect" denotes untreated infected mice, and "Uninfect" denotes uninfected and untreated mice. Data are presented as mean ± s.d., n = 5, "****" denotes p < 0.0001, "ns" denotes p > 0.05, One-way ANOVA with Tukey's post-hoc. P < 0.001, F = 32.26. Unless otherwise specified, pairwise p values from Tukey's post-hoc analysis are reported as "X, Y", where X represents comparison to "infect", and Y represents comparison to "uninfect".

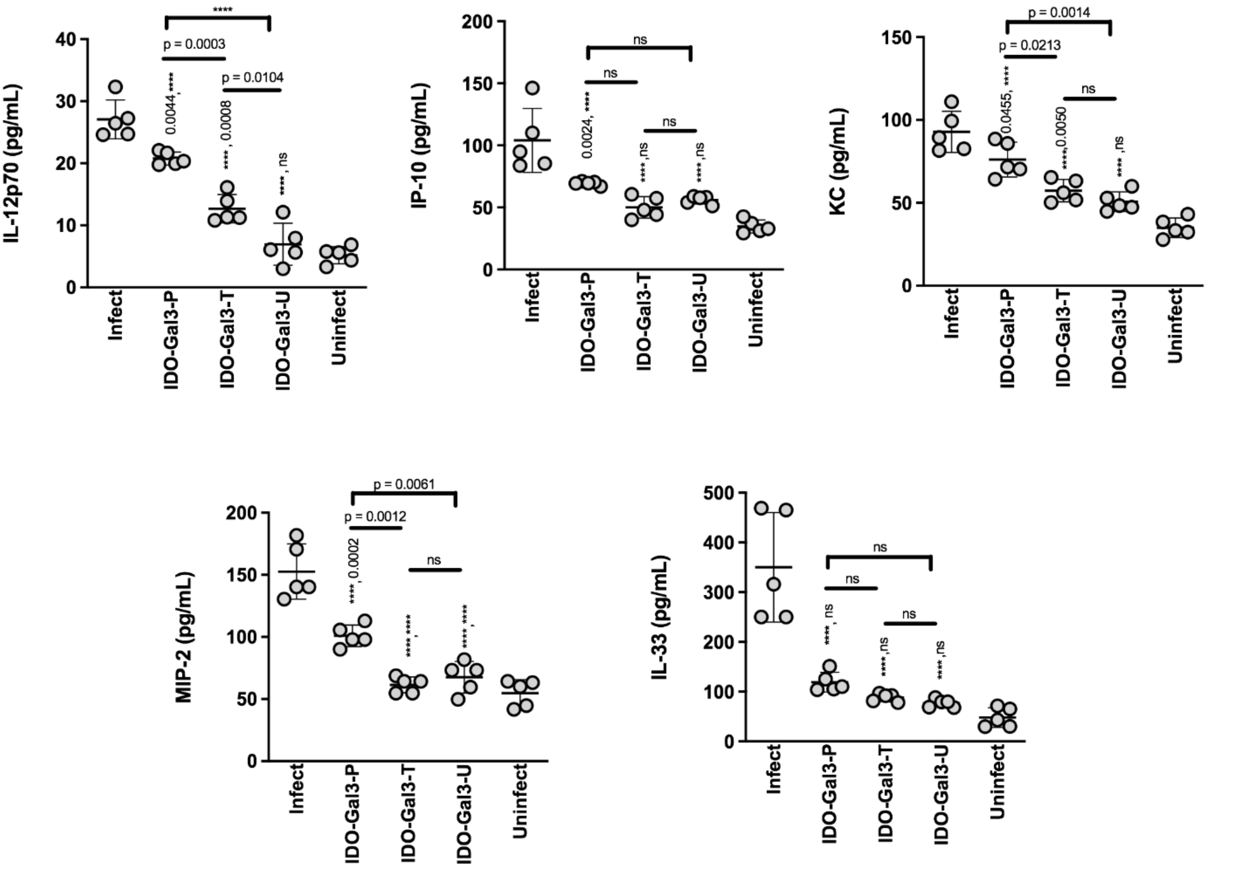

Supplementary Fig. 10 | Local cytokine expression following prophylactic (IDO-Gal3-P) or therapeutic (IDO-Gal3-T) submandibular injection of IDO-Gal3 in mice infected with PG and AA. Both prophylactic and therapeutic administration of IDO-Gal3 reduced gingival inflammatory protein levels of IL12p70, IP10, KC, MIP2 and IL33 with more pronounced effect following therapeutic administration. "IDO-Gal3-U" denotes animals that received IDO-Gal3 but were not infected, "Infect" denotes untreated infected mice, and "Uninfect" denotes uninfected and untreated mice. Data are presented as mean ± s.d., n = 5, "****" denotes $p < 0.0001$, "ns" denotes $p > 0.05$, One-way ANOVA with Tukey's post-hoc. For IL-12p70, $p < 0.0001$, $F = 72.92$. For IP-10, $p < 0.0001$, $F = 21.98$. For KC, $p < 0.0001$, $F = 33.32$. For MIP-2, $p < 0.0001$, $F = 46.46$. For IL-33, $p < 0.0001$, $F = 28.57$. Unless otherwise specified, pairwise p values from Tukey's post-hoc analysis are reported as "X, Y", where X represents comparison to "infect", and Y represents comparison to "uninfect".

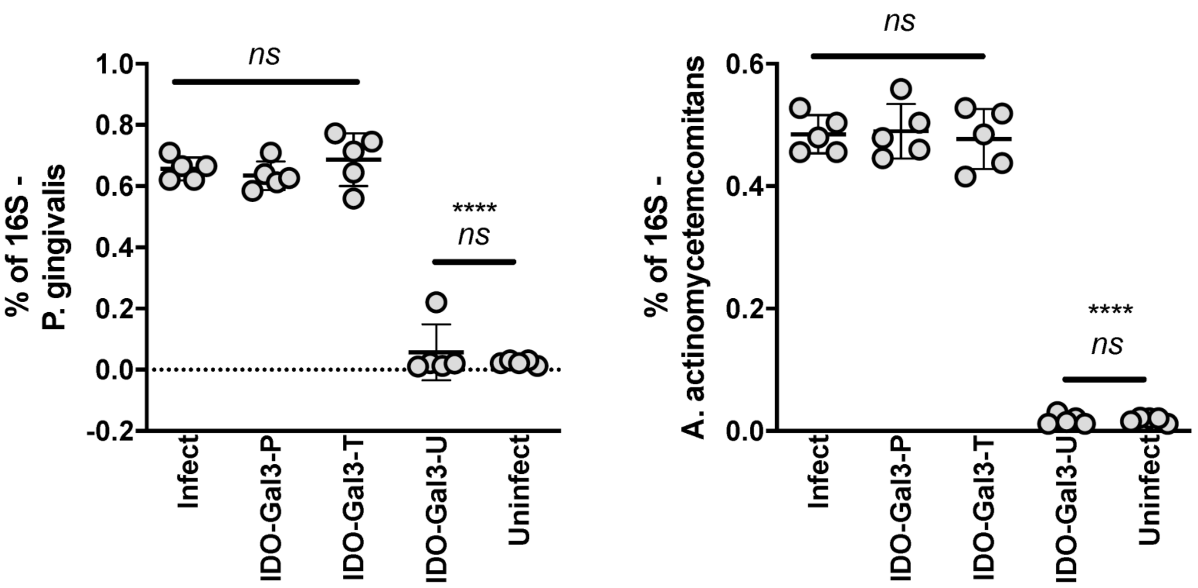

Supplementary Fig. 11 I Bacterial load in infected mice that received a prophylactic (IDO-Gal3-P) or therapeutic (IDO-Gal3-T) submandibular injection of IDO-Gal3. Recovery of *P. gingivalis* and *A. actinomycetemcomitans* from the oral cavity as measured by qPCR using 18s specific primers. Data are expressed as a % of total bacteria recovered as measured by qPCR using 18s consensus primers. Neither prophylactic (IDO-Gal3-P) nor therapeutic (IDO-Gal3-T) administration of IDO-Gal3 affects the % of *P. gingivalis* and *A. actinomycetemcomitans* recovered from the oral cavity. “IDO-Gal3-U” denotes animals that received IDO-Gal3 but were not infected, “Infect” denotes untreated infected mice, and “Uninfect” denotes uninfected and untreated mice. Data are presented as mean ± s.d., n = 5, “ns” indicates no difference between indicated groups, **** indicates $p < 0.0001$ compared to “infect” group, one-way ANOVA with Tukey’s post-hoc. For P. gingivalis, $p < 0.0001$, $F = 149.2$. For A. actinomycetemcomitans, $p < 0.0001$, $F = 297.9$.

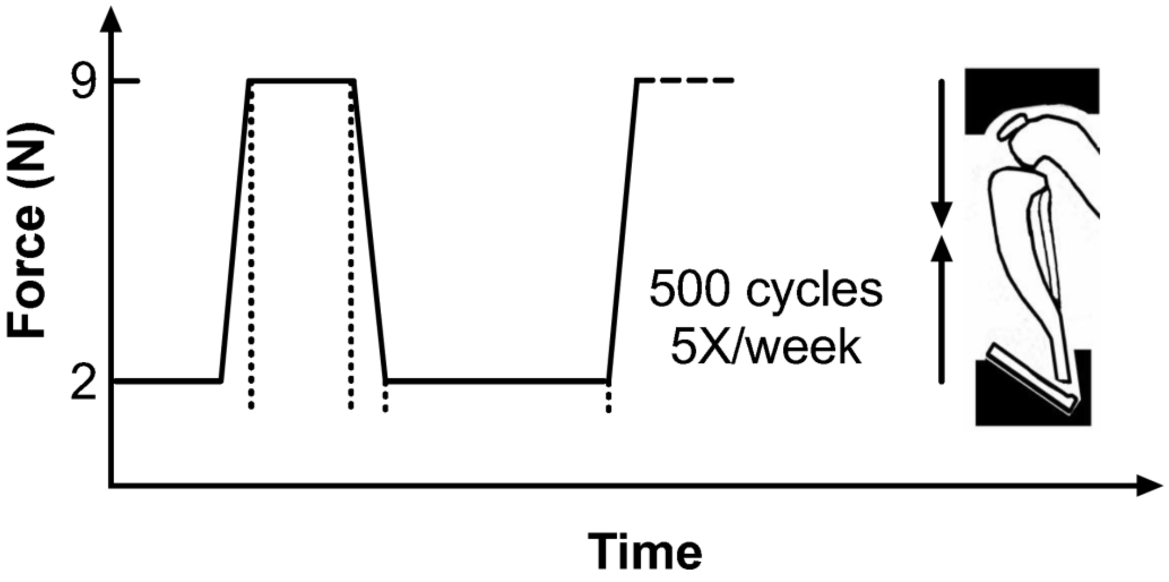

Supplementary Fig. 12 l Graphical representation of the mechanical over-loading protocol used to induce murine osteoarthritis.

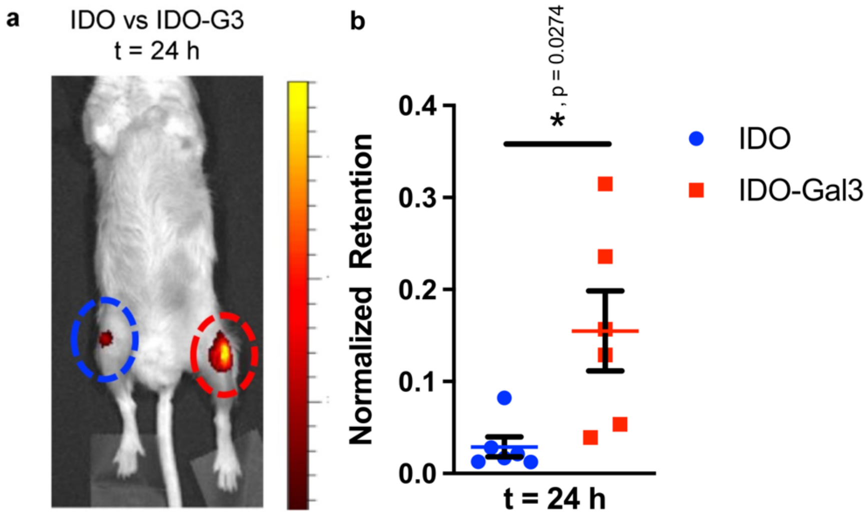

Supplementary Fig. 13 l (a) Representative image of IDO (blue, left) and IDO-Gal3 (red, right) retained within the mouse knee 24 h after injection. (b) Fraction of initial fluorescence signal retained at the injection site 24 h after injection. Data represented as mean ± s.e.m., n = 6, * represents $p < 0.05$, two-tailed Student's t-test, t = 3.083, df = 5.

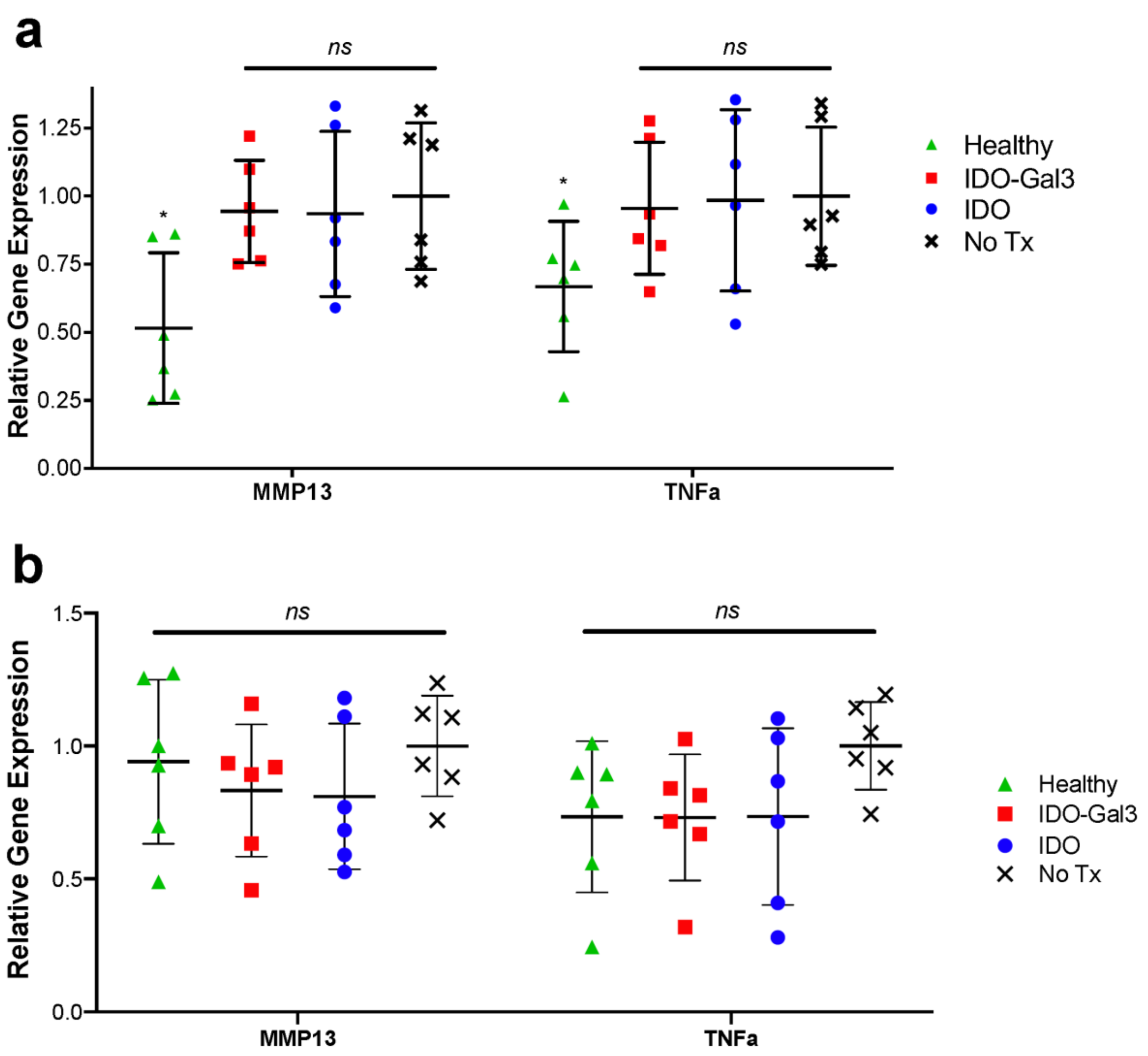

Supplementary Fig. 14 l Gene expression in (a) the knee with mechanically induced osteoarthritis and (b) the popliteal lymph node. Data presented as mean ± s.d., n = 6, * denotes $p < 0.05$ relative to all other groups, "ns" denotes no difference between indicated groups, Brown-Forsythe and Welch ANOVA with Tukey's post-hoc. For (a) MMP13, p = 0.0176, F* = 4.358. For (a) TNFa, p = 0.1448, F* = 2.032. For (b) MMP13, p = 0.5515, F* = 0.7225. For (b) TNFa, p = 0.2363, F* = 1.560.

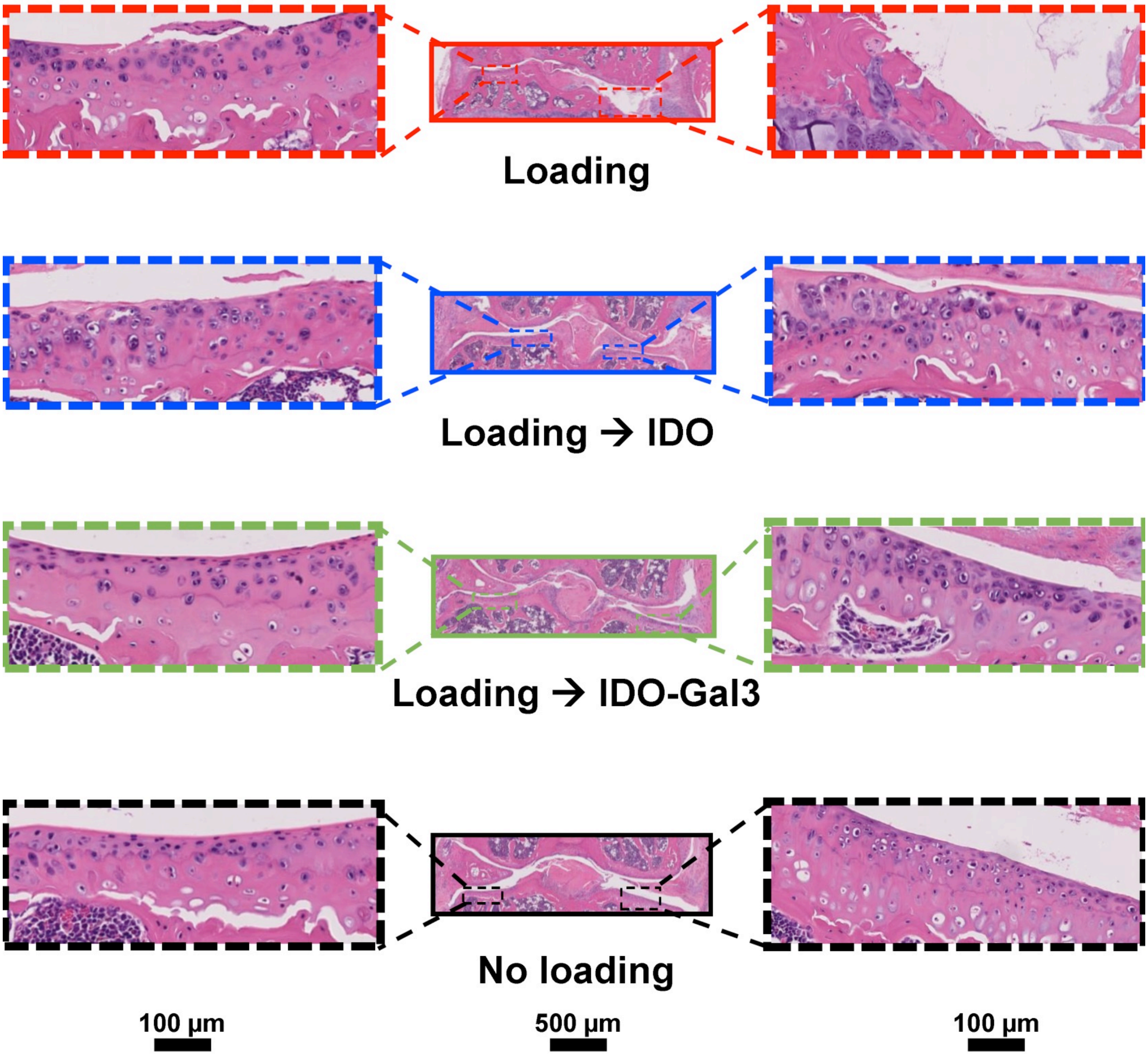

Supplementary Fig. 15 I Representative Hematoxylin & Eosin staining of whole joints and tibial articular cartilage surfaces per treatment group shown as a serial magnification.

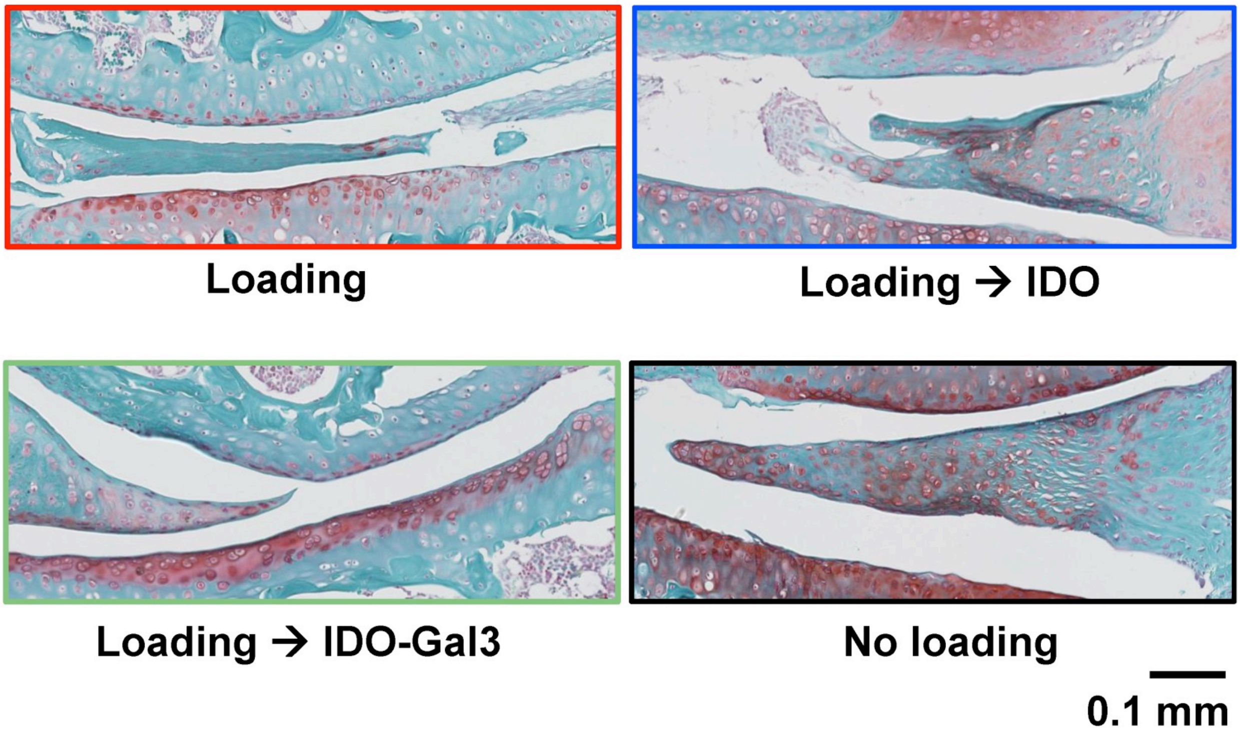

Supplementary Fig. 16 I Representative Safranin-O/Fast Green staining of the femoral-tibial cartilage interface with accompanying meniscus per treatment group.

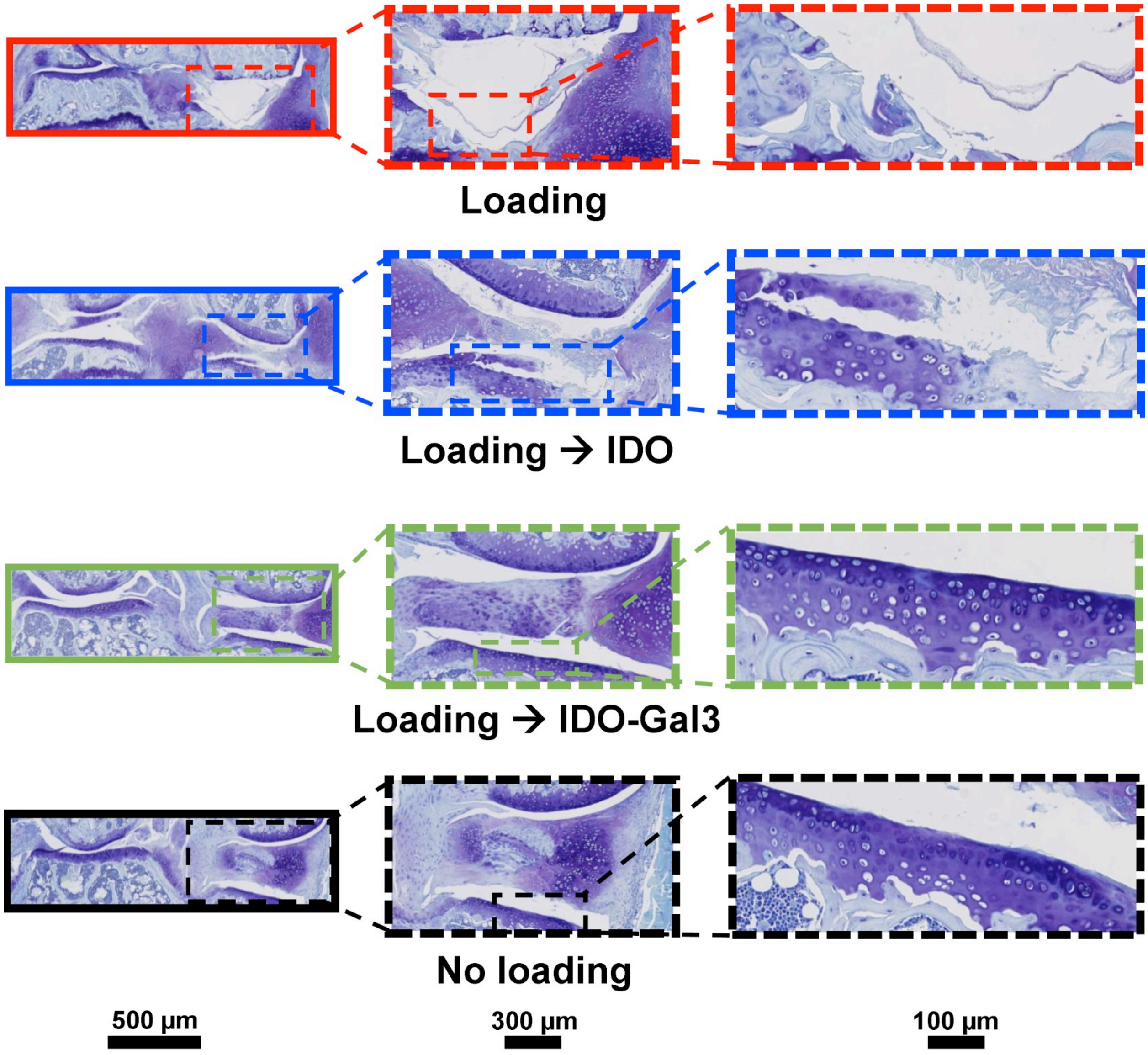

Supplementary Fig. 17 I Representative Toluidine Blue staining of whole joints, half joints, and tibial articular cartilage surfaces per treatment group shown as a serial magnification.

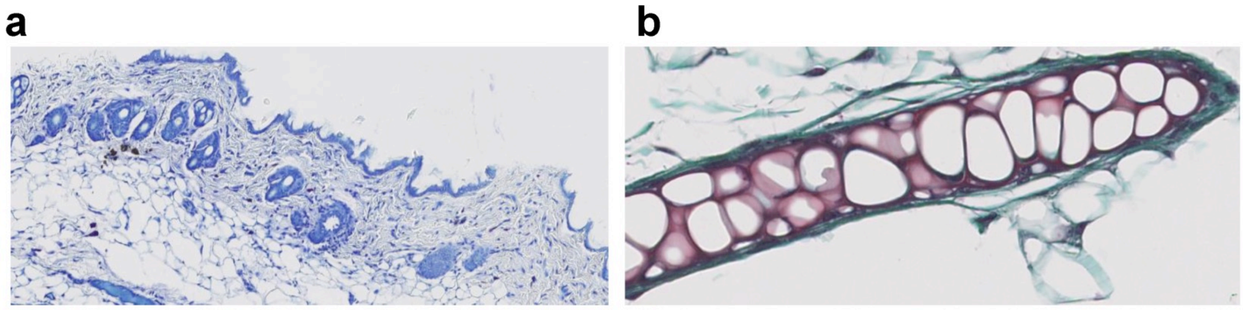

Supplementary Fig. 18 l (a) Toluidine staining control of mouse skin containing mast cells. (b) Safranin-O/Fast green staining control of mouse ear tissue.

Severity Score	DJD Scale
1	Moderate: articular cartilage degeneration with beginning of secondary pathology, including synovitis, joint capsule fibrosis, meniscal metaplasia
2	Marked: metaplasia and/or fragmentation of one meniscus, osteophyte formation, synovitis/hyperplasia
3	Severe: metaplasia and/or fragmentation of both menisci, total loss of at least 1 articular surface (eburnation), osteophyte formation, advanced synovitis/hyperplasia

Supplementary Fig. 19 l Overview of the DJD scoring rubric.

Severity Score	OARSI Scale
0	Normal
1	Loss of SO staining, thinning of articular cartilage; no defects
2	1 + fibrillation or pyknotic articular chondrocytes
3	2 + loss of articular cartilage <50% (e.g. erosion, flap, or callus)
4	3 + fragmentation and fissuring in an area<br>4 + fragmentation and fissuring in >75% of the cartilage surface
5	Total loss of normal articular cartilage (end-stage)
6	Loss of SO staining, thinning of articular cartilage; no defects

Supplementary Fig. 20 l Overview of the OARSI scoring rubric.

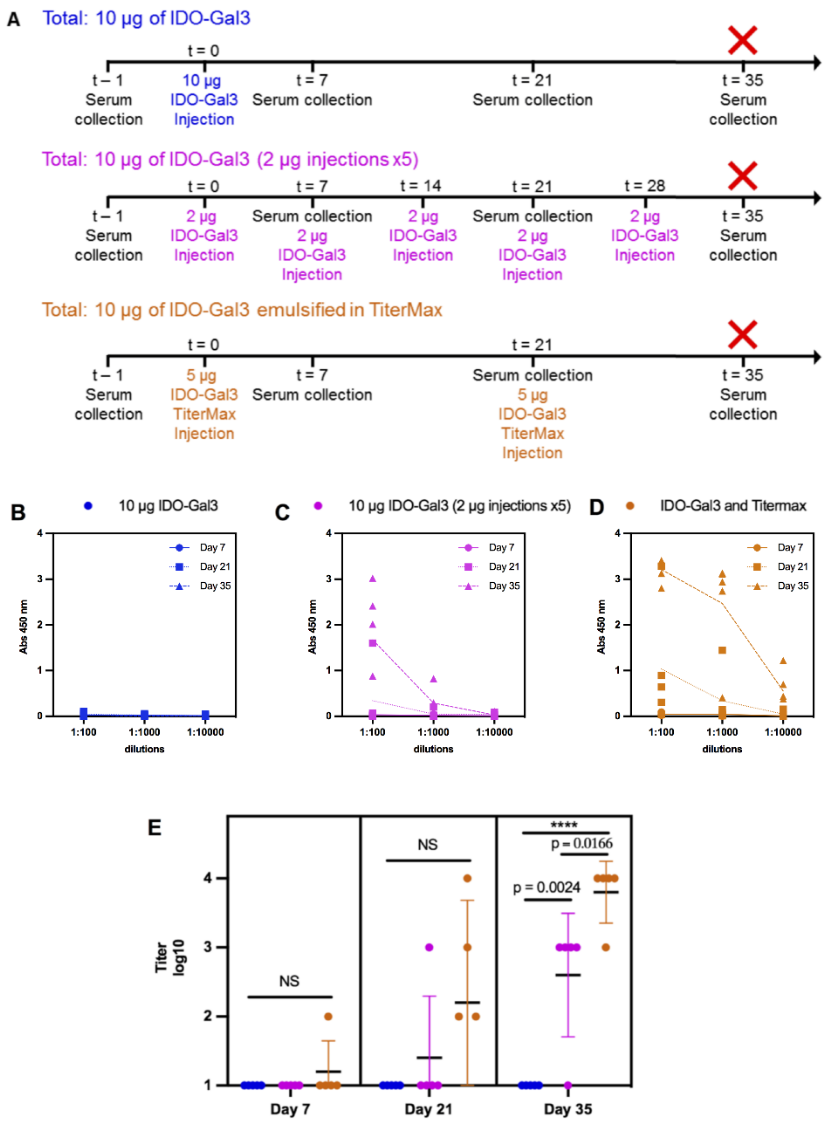

Supplementary Fig. 21. IDO-Gal3 immunogenicity in C57BL/6J mice. A) Injection and sample collection schedule for animals that received one injection of IDO-Gal3, multiple injections of IDO-Gal3, or IDO-Gal3 emulsified in TiterMax® adjuvant. (B-D) Total serum IgG reactive against IDO-Gal3 at day 7, day 21, and day 35. E) Positive titers are considered when the absorbance at day 7, day 21, and day 35 is higher than the absorbance average plus 5x standard deviation at day 0. Data presented as mean ± standard deviation (n = 5) in (B-D). Data presented as mean ± standard deviation (n = 5) in (E). **** represents $p < 0.0001$ ANOVA with Tukey's post-hoc.

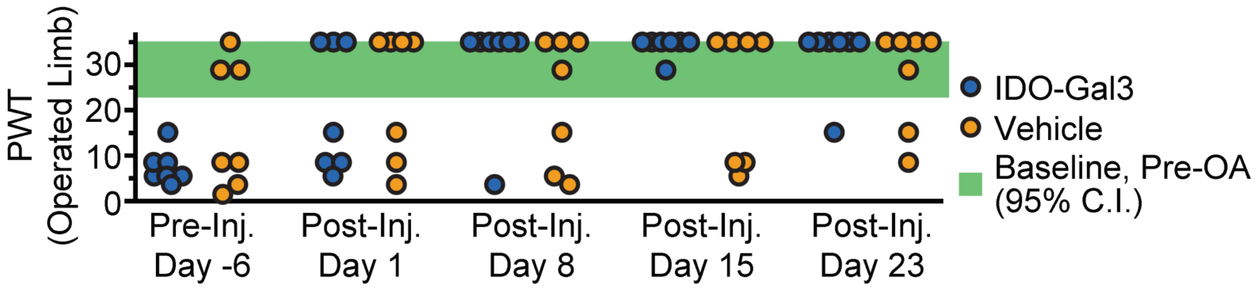

Supplementary Fig. 22. Paw withdrawal thresholds (PWT) where the animal is equally likely to tolerate versus withdraw from on touch stimulus.

a)

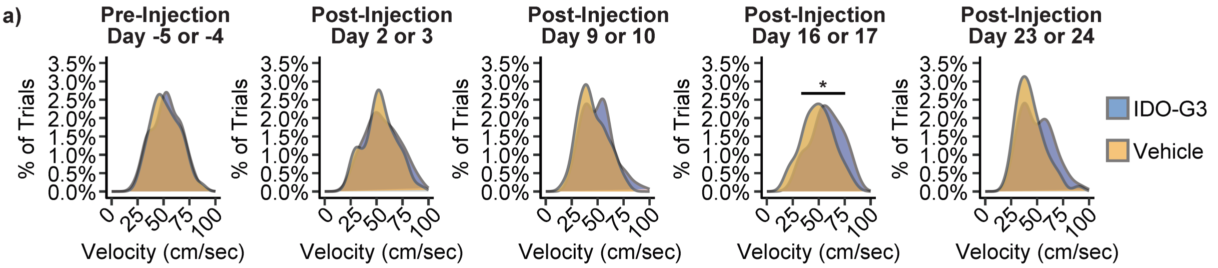

b)

	Pre-Injection Day -5 or -4		Post-Injection Day 2 or 3		Post-Injection Day 9 or 10		Post-Injection Day 16 or 17		Post-Injection Day 23 or 24	
	n	mean (95% C.I.)	n	mean (95% C.I.)	n	mean (95% C.I.)	n	mean (95% C.I.)	n	mean (95% C.I.)
IDO-G3	122	52.6 (50.1, 55.1)	121	55.8 (52.7, 58.8)	118	49.9 (47.1, 52.6)	102	57.6 (54.5, 60.6)	131	49.9 (47.2, 52.7)
Saline	128	52.3 (49.9, 54.7)	106	53.4 (50.5, 56.4)	118	45.5 (42.7, 47.7)	102	49.4 (46.4, 52.4)	113	43.8 (41.2, 46.3)

c)

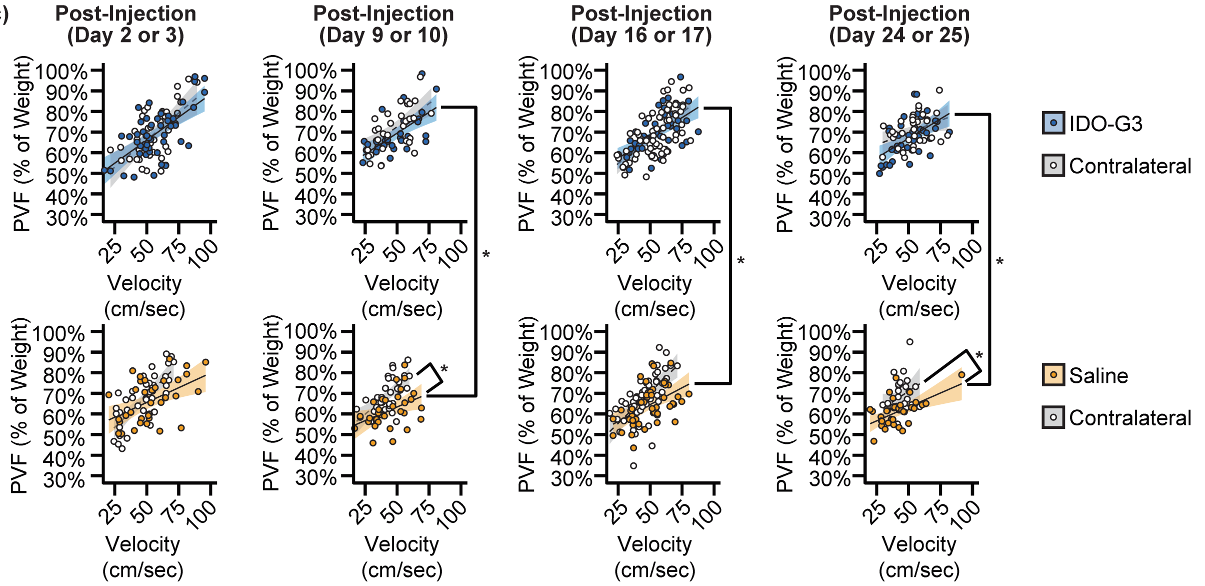

Supplementary Fig. 23. Distribution of selected walking velocity (a) along with trial counts, mean, and 95% confidence intervals (b). IDO-Gal3-treated animals used faster walking velocities at post-injection day 16 ($p=0.02$) and tended to use faster velocities at post-injection day 23 ($p=0.051$). (c) Raw data of the peak vertical force-time relationships used to project 95% confidence intervals for IDO-Gal3- and saline-treated knees, along with their respective contralateral controls. Error bands represent 95% C.I. All p-values associated with "*" are reported in Fig. 4.
