# Supplementary material for: Suppression of local inflammation via galectin-anchored indoleamine 2,3-dioxygenase
Source: Nat Biomed Eng. 2023 May 1;7(9):1156–69. doi: 10.1038/s41551-023-01025-1 (PMC10504068; doi:10.1038/s41551-023-01025-1)
Supplement: Supplementary file 1 [file 41551_2023_1025_MOESM2_ESM.pdf]

nature portfolio

Corresponding author(s): Gregory Hudalla and Benjamin Keselowsky

Last updated by author(s): Mar 15, 2023

Reporting Summary

Nature Portfolio wishes to improve the reproducibility of the work that we publish. This form provides structure for consistency and transparency in reporting. For further information on Nature Portfolio policies, see our Editorial Policies and the Editorial Policy Checklist.

Statistics

For all statistical analyses, confirm that the following items are present in the figure legend, table legend, main text, or Methods section.

n/a	Confirmed	
☐	☒	The exact sample size (*n*) for each experimental group/condition, given as a discrete number and unit of measurement
☐	☒	A statement on whether measurements were taken from distinct samples or whether the same sample was measured repeatedly
☐	☒	The statistical test(s) used AND whether they are one- or two-sided<br>*Only common tests should be described solely by name; describe more complex techniques in the Methods section.*
☐	☒	A description of all covariates tested
☐	☒	A description of any assumptions or corrections, such as tests of normality and adjustment for multiple comparisons
☐	☒	A full description of the statistical parameters including central tendency (e.g. means) or other basic estimates (e.g. regression coefficient) AND variation (e.g. standard deviation) or associated estimates of uncertainty (e.g. confidence intervals)
☐	☒	For null hypothesis testing, the test statistic (e.g. *F*, *t*, *r*) with confidence intervals, effect sizes, degrees of freedom and *P* value noted<br>*Give P values as exact values whenever suitable.*
☒	☐	For Bayesian analysis, information on the choice of priors and Markov chain Monte Carlo settings
☒	☐	For hierarchical and complex designs, identification of the appropriate level for tests and full reporting of outcomes
☒	☐	Estimates of effect sizes (e.g. Cohen's *d*, Pearson's *r*), indicating how they were calculated

Our web collection on statistics for biologists contains articles on many of the points above.

Software and code

Policy information about availability of computer code

Data collection	Matlab 2020b, Leica SCN400 Slide Scanner, Applied Biosystems QuantStudio 12K Flex Real-Time PCR System (with Built-In Software), IVIS Spectrum In Vivo Imaging System (PerkinElmer), Luminex 200 system with xPNONENT 3.1 software; NRecon and Dataviewer software (Skyscan); software: Living Image version 4.7.4and4.3.l; Phantom Micro Lab320 (Phantom Camera Control 3.0).
Data analysis	Graphpad Prism 8 and 9; R Analytics 4.0.4 with RStudio 2022; xPONENT 3.1 software; GAITORSuiteand GEKO custom codes are available at https:// github.com/OrthoBME, https://github.com/OrthoBME/GAITORsuite and https://github.com/OrthoBME/GEKO

For manuscripts utilizing custom algorithms or software that are central to the research but not yet described in published literature, software must be made available to editors and reviewers. We strongly encourage code deposition in a community repository (e.g. GitHub). See the Nature Portfolio guidelines for submitting code & software for further information.

Data

Policy information about availability of data

All manuscripts must include a data availability statement. This statement should provide the following information, where applicable:

- Accession codes, unique identifiers, or web links for publicly available datasets
- A description of any restrictions on data availability
- For clinical datasets or third party data, please ensure that the statement adheres to our policy

The main data supporting the results in this study are available within the paper and its Supplementary Information. The source datasets are available for research purposes from the corresponding authors on reasonable request.

Human research participants

Policy information about studies involving human research participants and Sex and Gender in Research.

Reporting on sex and gender	The study did not involve human research participants.
Population characteristics	—
Recruitment	—
Ethics oversight	—

Note that full information on the approval of the study protocol must also be provided in the manuscript.

Field-specific reporting

Please select the one below that is the best fit for your research. If you are not sure, read the appropriate sections before making your selection.

☒ Life sciences ☐ Behavioural & social sciences ☐ Ecological, evolutionary & environmental sciences

For a reference copy of the document with all sections, see nature.com/documents/nr-reporting-summary-flat.pdf

Life sciences study design

All studies must disclose on these points even when the disclosure is negative.

Sample size	Sample sizes were determined via power analysis (a = 0.05, power = 80%) of historical data from similar experiments or from pilot experiments for new studies, as per Rosner B. Fundamentals of Biostatistics. 7th ed. Boston, MA: BrooksLCole; 2011.
Data exclusions	No data were excluded.
Replication	The experimental compound reported here, IDO-Gal3, was evaluated for anti-inflammatory activity in multiple different animal models, by multiple different investigators, at multiple different institutions. All replication attempts were successful.
Randomization	Mice were randomly allocated, by housing cage, to experimental or control groups upon receipt. Rats with surgically induced OA were allocated into treatment and control groups after baseline measurement of disease severity.
Blinding	A blinded researcher evaluated each dataset included in the study.

Reporting for specific materials, systems and methods

We require information from authors about some types of materials, experimental systems and methods used in many studies. Here, indicate whether each material, system or method listed is relevant to your study. If you are not sure if a list item applies to your research, read the appropriate section before selecting a response.

Materials & experimental systems

n/a	Involved in the study
☐	☒ Antibodies
☒	☐ Eukaryotic cell lines
☒	☐ Palaeontology and archaeology
☐	☒ Animals and other organisms
☒	☐ Clinical data
☒	☐ Dual use research of concern

Methods

n/a	Involved in the study
☒	☐ ChIP-seq
☒	☐ Flow cytometry
☒	☐ MRI-based neuroimaging

Antibodies

Antibodies used	Commercial antibodies were used: biotinylated antibodies for CTXII (cat. # AC-08F1, ImmunoDiagnostic Systems, Copenhagen, Denmark), ILG and MCPI (cat.# 517703 and 505908, Biolegend, San Diego, CA, USA).
Validation	The antibodies were validated by the manufacturers, as described on their websites. The antibodies were further validated by using positive and negative populations versus isotype control antibodies.

Animals and other research organisms

Policy information about studies involving animals; ARRIVE guidelines recommended for reporting animal research, and Sex and Gender in Research

Laboratory animals	12-weeks-old female C57BL/Gj mice, and 12–14-weeks-old male Lewis rats.
Wild animals	The study did not involve wild animals.
Reporting on sex	Sex-based analyses were not performed.
Field-collected samples	The study did not involve samples collected from the field.
Ethics oversight	UF IACUC and Vanderbilt University IACUC.

Note that full information on the approval of the study protocol must also be provided in the manuscript.
